# PGC-1α repression dysregulates lipid metabolism and induces lipid droplet accumulation in the retinal pigment epithelium

**DOI:** 10.1038/s41419-024-06762-y

**Published:** 2024-06-01

**Authors:** Shuyan Zhou, Kaan Taskintuna, Jacob Hum, Jasmine Gulati, Stephanie Olaya, Jeremy Steinman, Nady Golestaneh

**Affiliations:** 1 Department of Ophthalmology, Georgetown University Medical Center, Washington, DC 20007 USA; 2 Department of Neurology, Georgetown University Medical Center, Washington, DC 20007 USA; 3 Department of Biochemistry and Molecular & Cellular Biology, Washington, DC 20007 USA

**Keywords:** Mechanisms of disease, Fatty acids

## Abstract

Drusen, the yellow deposits under the retina, are composed of lipids and proteins, and represent a hallmark of age-related macular degeneration (AMD). Lipid droplets are also reported in the retinal pigment epithelium (RPE) from AMD donor eyes. However, the mechanisms underlying these disease phenotypes remain elusive. Previously, we showed that *Pgc-1α* repression, combined with a high-fat diet (HFD), induce drastic AMD-like phenotypes in mice. We also reported increased PGC-1α acetylation and subsequent deactivation in the RPE derived from AMD donor eyes. Here, through a series of in vivo and in vitro experiments, we sought to investigate the molecular mechanisms by which *PGC-1α* repression could influence RPE and retinal function. We show that PGC-1α plays an important role in RPE and retinal lipid metabolism and function. In mice, repression of *Pgc-1α* alone induced RPE and retinal degeneration and drusen-like deposits. In vitro inhibition of *PGC1A* by CRISPR-Cas9 gene editing in human RPE (ARPE19- *PGC1A* KO) affected the expression of genes responsible for lipid metabolism, fatty acid β-oxidation (FAO), fatty acid transport, low-density lipoprotein (LDL) uptake, cholesterol esterification, cholesterol biosynthesis, and cholesterol efflux. Moreover, inhibition of *PGC1A* in RPE cells caused lipid droplet accumulation and lipid peroxidation. ARPE19-*PGC1A* KO cells also showed reduced mitochondrial biosynthesis, impaired mitochondrial dynamics and activity, reduced antioxidant enzymes, decreased mitochondrial membrane potential, loss of cardiolipin, and increased susceptibility to oxidative stress. Our data demonstrate the crucial role of PGC-1α in regulating lipid metabolism. They provide new insights into the mechanisms involved in lipid and drusen accumulation in the RPE and retina during aging and AMD, which may pave the way for developing novel therapeutic strategies targeting PGC-1α.

## Introduction

Age-related macular degeneration (AMD) is the leading cause of blindness in the elderly. Currently, more than 11 million Americans and over 200 million people worldwide are affected by AMD, and its prevalence is expected to double by 2050 [1]. AMD is a multifactorial disease in which aging is the major factor; however, a complex interplay between genetic, environmental, and metabolic factors contributes to the disease etiology [2].

Drusen, a hallmark of dry AMD, consist of extracellular deposits, which accumulate between the RPE and Bruch’s membrane (BM) [3]. Lipid droplet and lipofuscin accumulations have also been reported in the aging RPE and RPE derived from AMD donor eyes [4].

Several studies have reported on mitochondrial dysfunction, reduced mitochondrial activity, and a switch from oxidative phosphorylation to anaerobic glycolysis, resembling Warburg syndrome in AMD [5–7].

Mitochondrial biogenesis is regulated by peroxisome proliferator-activated receptor γ coactivator 1α (PGC-1α), which regulates mitochondrial mass through induction of transcriptional machinery [8] and is involved in carbohydrate and lipid metabolism [9].

PGC-1α is reported to influence fatty acid oxidation (FAO) by upregulating the expression of tricarboxylic acid cycle genes of the mitochondrial fatty acid oxidation pathway [10].

In addition, stimulation of PGC-1α has been shown to upregulate the plasma membrane and mitochondrial fatty acid transporters (FAT/CD36, FABPpm, and FATPs) content in skeletal muscle [11].

Lipids can be contained in cells as lipid droplets, which are storage organelles containing neutral lipids and originate from the endoplasmic reticulum (ER). The size, composition, and number of lipid droplets differ between cells and could reflect cellular metabolic states [12]. Accumulation of lipid droplets has also been related to mitochondrial dysfunction as a generalized response to oxidative stress [13].

PGC-1α is also shown to regulate mitochondrial membrane structure and function by metabolic and membrane structural programs in the heart [14].

An important component of mitochondrial membranes is Cardiolipin (CL), which is exclusively synthesized in mitochondria [15]. CL plays an essential role in mitochondrial functions, including mitochondrial coupling and energy production, and its synthesis has been shown to be regulated by PGC-1α in the heart [14, 16].

Studies have reported on the role of PGC-1α in RPE oxidative metabolism and antioxidant defense [17], retinal light sensitivity [18], regulation of normal and pathological retinal angiogenesis [19], and induction of mesenchymal transition in RPE and retinal degeneration [20].

Previously, we showed that heterozygous PGC-1α repression (*Pgc-1*α^*+/−*^) combined with a high-fat diet (HFD) induces AMD-like phenotypes in mice [21]. While a HFD exacerbates lipid accumulation and accelerates induction of disease phenotypes in the RPE and retina of the *Pgc-1*α^*+/−*^ mice, as shown by our previous study, we demonstrate here that inhibition of *Pgc-1*α alone, under a regular diet, can induce RPE and retinal dysfunction and drusen-like deposits in mice.

We also reported dysregulated AMPK-SIRT1-PGC-1α and metabolic pathways [22] and lipid accumulation in the RPE of AMD donor eyes [6]. A recent study also showed that lipid droplet accumulation can induce RPE dysfunction [23].

However, the mechanisms underlying these cellular dysfunctions remain to be elucidated.

Here, we demonstrate the critical roles of PGC-1α in regulating lipid metabolism in RPE. We also show that inhibition of *PGC1A* in human RPE reduces the expression of genes related to lipid metabolism, inhibits β-oxidation, represses triglyceride synthesis while promoting cholesteryl ester generation, induces lipid droplet accumulation, reduces mitochondrial mass and function, decreases antioxidant enzymes, and thus, increases the susceptibility of the RPE to oxidative stress-induced cell death by peroxidation.

Our data shed light on the mechanisms of lipid accumulation and metabolic dysfunction in RPE and retina and open new avenues for developing novel treatment strategies in AMD.

## Materials and methods

### Cells

Human RPE (ARPE-19) cells were obtained and validated as previously described [24]. ARPE19 cells were cultured daily in DMEM/F12 medium (11320033; Life Technologies, Carlsbad, CA, USA) supplemented with 10% FBS (10438-026, Life Technologies) and 2 mM GlutaMAX (35650061, Life Technologies).

ARPE19-*PGC1A* KO cells were generated by CRISPR/Cas9-mediated technology. Specific guide RNAs (gRNAs) were designed using the following link: https://zlab.bio/guide-design-resources and ligated into LentiCRISPR v2 (TLCV2) according to Addgene’s protocol. TLCV2 was a gift from Adam Karpf (Addgene plasmid # 87360; http://n2t.net/addgene:87360 ; RRID:Addgene_87360). The gRNAs used in this study are as follows: *PGC1A* sgRNA1 (5′–3′): GATACAGACAGCTTTCTGGG, *PGC1A* sgRNA2 (5′–3′): GCTTTCTGGGTGGACTCAAG. Cells transfected with Control (Scramble) or sgRNAs were selected in complete medium with 2 μg/ml puromycin for 2 weeks, followed by adding 2 μM doxycycline (Dox) for 24 h to induce Cas9-eGFP expression. The ratio of Cas9 positive cells was analyzed by flow cytometry at 48 h post Dox induction. ARPE19-*PGC1A* KO and Scramble control cells were cultured in complete medium with 2 μg/ml puromycin and 2 μM Dox for 2 weeks to effectively repress *PGC1A*. Dox was then removed and cells were cultured for an additional week to conduct qPCR and mtDNA copy number assay. Cells deprived of Dox were cultured for two additional weeks to perform Western Blotting, L-Carnitine assay, Fatty acid oxidation assay, lipid peroxidation assay, TMRE-mitochondrial membrane potential assay, mitochondrial staining, and cell viability assay.

Two different si*PGC1As*: siRNA1 (5′–3′): CAUUUGAGAACAAGACUAU, siRNA2(5′–3′): GAAUUCAUGGAGCAAUAAA, and siControl (5′–3′): AACGUACGCGGAAUACUUCGA were synthesized by Sigma Aldrich and packed by Lipofectamine RNAiMAX Transfection Reagent (13778075, Thermo Fisher Scientific, Waltham, MA, USA), and delivered to ARPE19 cells in Opti-MEM reduced serum media (31985070, Life Technologies). Cells were cultured 4–6 h followed by incubation in complete media. Cells were collected in 72 h for qPCR and western blots.

### Animals

All studies involving animals were approved by the Institutional Animal Care and Use Committee of Georgetown University and were in compliance with the Statement for the Use of Animals in Ophthalmic and Vision Research from the Association for Research in Vision and Ophthalmology (ARVO). Heterozygous B6.129S4 (FVB)-Ppargc1a^tm1Brsp^/J mice on a C57BL/6J background purchased from the Jackson Laboratory (Bar Harbor, ME, USA) were bred to generate *Pgc-1α*^+/−^ and *Pgc-1α*^−/−^ mice. Animals were kept in a temperature-controlled room on a 12-h dark:12-h light cycle with free access to water and food. Automated genotyping from tail samples was performed by Transnetyx (Cordova, TN, USA). The sample size was chosen based on between-subject variability for each experimental group for in vivo experiments. Mice from both sexes were included in the study. Mice were excluded from the study in case of any apparent pathology, adverse reactions to anesthesia, or abnormal behavior. Mice were randomly incorporated into experimental groups based on genotype. Experiments were performed in a blind and randomized manner.

### qRT-PCR

RNA was extracted from harvested cells using the E.Z.N.A Total RNA Kit I (6834-01; Omega Bio-Tek, Norcross, GA, USA). cDNA was synthesized using the Verso cDNA synthesis Kit (AB1453B; Thermo Fisher Scientific) and qPCR amplification was performed with the Maxima SYBR Green/ROX qPCR Master Mix (2X) (K0222, Thermo Fisher Scientific) by loading 10 ng cDNA for each reaction. qPCR primers (Table S1) were designed using PrimerBank (https://pga.mgh.harvard.edu/primerbank/) and synthesized by Integrated DNA Technologies (Coralville, IA, USA).

### Western Blotting

For whole-cell protein extraction, collected cells were lysed by T-PER tissue protein extraction reagent (78510, Thermo Fisher Scientific) using protease and phosphatase inhibitor (A32959, Thermo Fisher Scientific). For mitochondrial proteins, mitochondria were isolated with the mitochondria Isolation Kit (89874, Thermo Fisher Scientific). A BCA assay kit was used to determine protein concentration (23227, Thermo Fisher Scientific). 30μg protein samples for each lane were loaded into Tris-glycine gels (8%, 10%, or 12%), and proteins were transferred onto PVDF membranes (IPVH00005; MilliporeSigma, Burlington, MA, USA). Blots were incubated with the primary antibody overnight at 4 °C. The blots were then washed three times and incubated with the secondary antibody for 1 h at room temperature. Corresponding primary antibodies and secondary antibodies with their respective dilutions are listed in Table S2.

### l-carnitine assay

l-Carnitine levels were determined by l-Carnitine Quantification Assay Kit (ab83392; Abcam, Waltham, MA, USA). 1.4 × 10^6^ cells for each group were harvested and samples were set up in duplicates. The assay and analysis were performed according to the manufacturer’s protocol. l-Carnitine for each group was calculated using the methods provided by the manufacturer.

### Fatty acid oxidation (FAO) assay

FAO activity was measured on cell lysates using the fatty acid oxidation (FAO) assay kit (E-141; Biomedical Research Service, Buffalo, NY, USA). 1 × 10^6^ cells/group were harvested and lysed in 95 μl of 1× lysis solution. BCA protein assay was used to determine lysate protein concentration. The concentration of all samples was equalized to 1 μg/μl using lysis buffer. 20 μl lysate of each sample was added to a 96-well plate in duplicate. Samples were incubated in a 37 °C humidified incubator for 1 h. FAO activity was calculated using manufacturer guidelines.

### Lipid peroxidation (MDA) assay

Lipid peroxidation levels of ARPE19-*PGC1A* KO and Scramble control cells were measured by the Lipid Peroxidation (MDA) Assay Kit (ab118970, Abcam). The relative fluorescence units (RFU) were measured on a 96-well black-bottom flat plate (655086; Greiner Bio-One, Kremsmünster. AT) in duplicates for each sample at Ex/Em=532/553 nm (top read). The concentration of MDA for each group was calculated according to the manufacturer’s instructions.

### Lipids droplets staining

ARPE19-*PGC1A* KO and Scramble control cells were cultured in complete medium with 2 μg/ml puromycin and 2 μM doxycycline for 2 weeks. Cells were then cultured for 6 or 10 additional weeks in DMEM/F12 medium with 15% FBS and 2 μg/ml puromycin. Cells were seeded in 35 mm glass bottom confocal dish (81218-200; Ibidi, Gräfelfing, GER). **All the following steps need to be protected from light**. When cells reached 100% confluency, Lipid Spot 488 was diluted (70065; 1:1000; Biotium, Fremont, CA, USA) in complete medium and cells were incubated with the dye at 37 °C for 1 h. Cells were gently washed with PBS one time and fixed in 4% formaldehyde for 15 min at room temperature, and permeabilized with 0.1% Triton™ X-100 for 10 min. Cells were blocked with 5% BSA for 1 h at room temperature, flowed by 3 washes by PBS. Cells were incubated with ZO-1 Alexa Fluor® 594 conjugated antibody (339194, Thermo Fisher Scientific) at 5 µg/ml (1:100) in 1% BSA for 3 h at room temperature. Cell nuclei were stained with DAPI (0100-20; Southern Biotech, Birmingham, AL, USA). Images were acquired with an ECHO Revolve Microscope (ECHO, San Diego, CA, USA). Green fluorescence of lipid droplets in three random areas from each image were quantified by ImageJ to relatively quantify lipid droplets for each group.

### mtDNA copy number

Genomic DNA of RPE cells were extracted by the DNeasy Blood &Tissue Kit (69504; Qiagen, Venlo, NL). The mitochondrial DNA (mtDNA) gene *ND1* and the nuclear DNA (nDNA) gene *ACTB* were amplified by CFX Connect Real-Time PCR detection system (Bio-Rad, Hercules, CA, USA). The qPCR protocol was performed as previously described [24]. Cycle threshold values for each sample were analyzed to determine the relative DNA copy number by a ratio of mitochondrial DNA to genomic DNA.

### Mitochondrial staining

Cells were seeded in 35 mm glass bottom confocal dish (81218-200, Ibidi) for 24 h, 5000 cells/dish. Freshly prepared complete medium containing 100 nM MitoView^TM^ Green (70054, Biotium) and 10ug/ml Hoechst33342 (for nuclei staining; H3570, Thermo Fisher Scientific) was directly added to the cells. Cells were incubated for 30 min at 37 °C and protected from light. Images from living cells were captured by the ECHO Revolve Microscope.

### TMRE-mitochondrial membrane potential assay

ARPE19-*PGC1A* KO and Scramble control cells were seeded on a 96-well plate (3603; Corning, NY, USA), 0.85 × 10^5^ cells/well in triplicate were cultured for 12 h, and the TMRE- Mitochondrial Membrane Potential Assay was performed according to the manufacturer’s instructions (ab113852, Abcam, MA, USA). TMRE values were measured at Ex/Em = 549/575 nm (bottom read).

### Cell viability assay

Cells were seeded on a 96-well plate in triplicate for 48 h, at the density of 1.4 × 10^4^ cells/well (3603, Corning), followed by incubation with a range of H_2_O_2_ concentrations (0–1 mM) for 24 h. Cell viability was measured with PrestoBlue Cell viability Reagent (A13261; Invitrogen, Waltham, MA, USA). Fluorescence was read through the top at Ex/Em = 560 nm/590 nm.

### Cardiolipin assay

2 × 10^7^ cells from ARPE19-*PGC1A* KO group and Scramble group were collected to isolate the mitochondria using the Mitochondria Isolation Kit (89874, ThermoFisher Scientific). The concentration of mitochondrial lysate was determined by BCA assay kit (23227, ThermoFisher Scientific). 40μg mitochondrial protein for each group was loaded for the measurement of Cardiolipin content by Cardiolipin Assay kit (ab241036, Abcam, MA, USA). Cardiolipin concentrations were reported as nmol of Cardiolipin/ mg mitochondrial protein.

### Electroretinography

Mice were 8 months old at the time of analysis. Following overnight dark-adaptation, mice were anesthetized with 3.0% isoflurane. Under dim red light, they were transferred on a heating pad at 37 °C where 1.5% isoflurane was continuously delivered through a nose cone for maintenance. 2.5% Phenylephrine: 1% Tropicamide eye drops at a 2:1 ratio was used to induce mydriasis. Celeris stimulator-electrodes (Diagnosys LLC, Lowell, MA, USA) were positioned on each cornea. GenTeal (0.3% hypromellose; Alcon, Fort Worth, TX, USA) was used to both facilitate contact of the cornea with the electrodes and lubricate the eyes. ERG responses were recorded using the Diagnosys Celeris system and the Espion V6 software.

Scotopic flash ERGs were recorded using a white flash (6500 K) at seven ascending stimulus intensities ranging from 0.001–10.0 cd s/m^2^. Responses were an average of three sweeps. The a-wave was measured from the pre-stimulus baseline to the trough of the initial negative wave. The b-wave amplitude was measured at the highest positive peak following the trough of the a-wave. The c-wave was recorded at a stimulus intensity of 150.0 cd s/m^2^; c-wave amplitude was measured from the pre-stimulus baseline to the highest recorded peak. Implicit times were recorded as the duration after the flash onset and the time to peak.

### Fundus photography

Animals were prepared as described above. Fundus images were acquired on live animals using the OcuScience iVivo Funduscope (OcuScience, Henderson, NV, USA). Adobe Photoshop was used to process the images. Fundus images were analyzed by counting drusen-like deposits that appear as bright spots on the fundus. The analysis was carried out by three independent investigators. Number of deposits observed from both eyes were averaged for each animal.

### Statistical analysis

For statistical analysis of dark-adapted flash ERG series, two-way repeated measures ANOVA (factors: genotype and luminance) and *post hoc* Sidak correction for multiple comparisons was used. For the comparison of c-wave data across three genotypes, one-way ANOVA with *post hoc* Sidak correction was used. Responses from both eyes were averaged for each data point. Data met assumptions of normality as tested by the Shapiro-Wilk test and homogeneity of variance. For comparison of fundus photographs across three groups, data were log transformed and analyzed by one-way ANOVA with *post hoc* Sidak correction. Data are presented as mean ± SEM; GraphPad Prism 9.5.0 was used for analysis.

For in vitro experiments, two-tailed unpaired *t*-test was performed for comparison between two groups. Data are presented as mean ± SD or mean ± SEM from three independent experiments. Significance was considered when *P ≤ 0.05 and was indicated in the text as follows: *P ≤ 0.05, **P ≤ 0.01, ***P ≤ 0.001, ****P ≤ 0.0001.

## Results

### *PGC-1α* knockout and heterozygous mice exhibit impaired photoreceptor and RPE function and drusen-like deposits

**Fig. 1:**
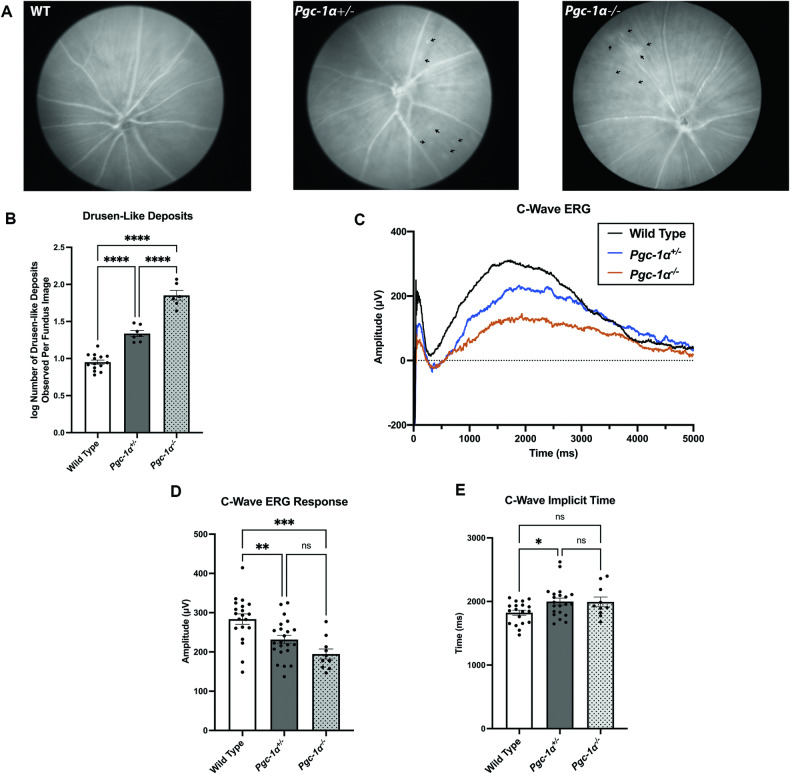
*Pgc-1α* heterozygous and knockout mice exhibit differences in phenotype and RPE function. **A** Representative monochromatic fundus images from 8-month-old Wild Type (WT), *Pgc-1α*^*+/−*^ (whole-body heterozygous), and *Pgc-1α*^*−/−*^ (whole-body knockout) mice. Arrows point to drusen-like deposits. **B** Comparison of the number of drusen-like deposits observed in the fundus field of view. WT (*n* = 14); *Pgc-1α*^*+/−*^ (*n* = 7); *Pgc-1α*^*−/−*^ (*n* = 6). **C** Representative RPE-generated c-wave ERG traces (*n* = 5) between WT, *Pgc-1α*^*+/−*^, and *Pgc-1α*^*−/−*^. **D** Quantification and comparison of RPE-generated c-wave amplitudes from WT (*n* = 20), *Pgc-1α*^*+/−*^ (*n* = 22), and *Pgc-1α*^*−/−*^ (*n* = 10) mice. **E** Quantification and comparison of c-wave implicit times (measured from start to peak amplitude) between WT, *Pgc-1α*^*+/−*^ and *Pgc-1α*^*−/−*^ mice. Both male and female mice were included in the study. Data were analyzed using one-way ANOVA followed by *post hoc* Sidak correction for multiple comparisons. Data are presented as mean ± SEM. *P ≤ 0.05, **P ≤ 0.01, ***P ≤ 0.001, ****P ≤ 0.0001, n.s. denotes no statistical significance.

In order to evaluate the overall ocular health of the *Pgc-1α*^*+/−*^ and *Pgc-1α*^−*/*−^, we performed fundus photography. Fundus images revealed drusen-like deposits in *Pgc-1α*^*+/−*^ and *Pgc-1α*^*−/−*^ as compared to WT mice. These deposits appeared to be bigger in size in the *Pgc-1α*^*−/−*^ compared to the *Pgc-1α*^*+/−*^ (Fig. 1A). Additionally, we observed a significantly higher number of drusen-like deposits in *Pgc-1α*^*+/−*^ and *Pgc-1α*^*−/−*^ mice compared to WT controls, as well as an increased amount in *Pgc-1α*^*−/−*^ vs. *Pgc-1α*^*+/−*^ (adj. *P* < 0.0001 for all three comparisons) (Fig. 1B).

Analysis of c-wave ERG revealed significantly reduced RPE function both in the *Pgc-1α*^*+/−*^ (adjusted ANOVA *P* = 0.0066) and *Pgc-1α*^*−/−*^ (adj *P* = 0.0002), compared to WT mice (Fig. 1C, D). Although we observed a trend towards reduced c-waves between *Pgc-1α*^*−/−*^ and *Pgc-1α*^*+/−*^, the difference was not statistically significant (Fig. 1D). c-wave implicit times were significantly delayed in *Pgc-1α*^*+/−*^ mice compared with WT controls (adj. *P* = 0.0409), and a trend was observed towards delayed c-wave responses in *Pgc-1α*^*−/−*^ mice as compared to WT controls; however, the difference was not statistically significant, probably due to a smaller sample size in *Pgc-1α*^*−/−*^ mice (Fig. 1E).

**Fig. 2:**
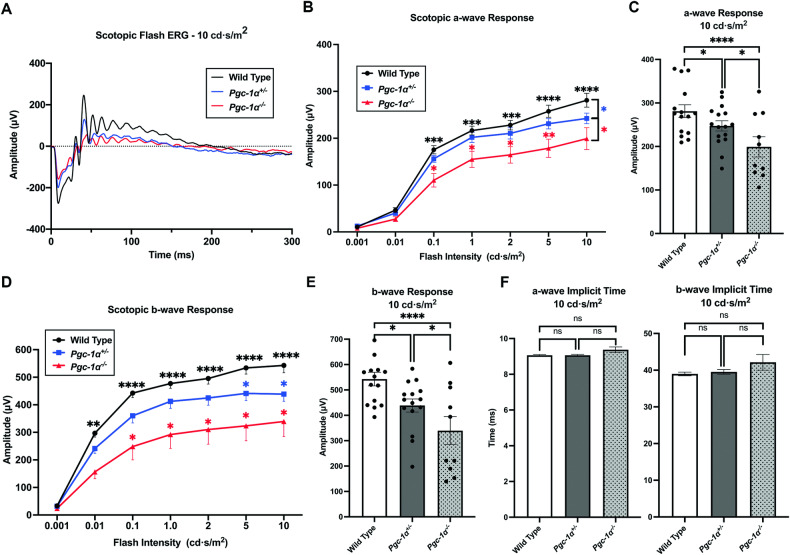
*Pgc-1α* repression leads to changes in photoreceptor and inner retinal function. **A** Representative scotopic flash ERG traces at the stimulus intensity of 10 cd s/m^2^ between WT, *Pgc-1α*^*+/−*^, and *Pgc-1α*^*−/−*^ mice (*n* = 5). **B** Line graph quantification of scotopic a-wave amplitudes between WT (*n* = 15), *Pgc-1α*^*+/−*^ (*n* = 15), and *Pgc-1α*^*−/−*^ (*n* = 10) mice at seven increasing stimulus intensities ranging from 0.001–10 cd s/m^2^. Black stars represent statistical significance between WT vs. *Pgc-1α*^*−/−*^*;* red represents *Pgc-1α*^*+/−*^ vs *Pgc-1α*^*−/−*^, and blue represents WT vs. *Pgc-1α*^*+/−*^. **C** Quantification of a-wave amplitudes at the stimulus intensity of 10 cd s/m^2^. **D** Line graph quantification of scotopic b-wave amplitudes between WT (*n* = 15), *Pgc-1α*^*+/−*^ (*n* = 15), and *Pgc-1α*^*−/−*^ (*n* = 10). Black stars represent statistical significance between WT *vs. Pgc-1α*^*−/−*^, red represents *Pgc-1α*^*+/−*^ *vs. Pgc-1α*^*−/−*^, and blue represents WT *vs. Pgc-1α*^*+/−*^. **E** Quantification of b-wave amplitudes at the stimulus intensity of 10 cd s/m^2^. **F** Quantification of a- and b-wave implicit times, respectively, at the stimulus intensity of 10 cd s/m^2^. Both male and female mice were included in the study. Two-way repeated measures ANOVA with *post hoc* Sidak correction for multiple comparisons was used for statistical analysis. Data are presented as mean ± SEM. *P ≤ 0.05, **P ≤ 0.01, ***P ≤ 0.001, ****P ≤ 0.0001, n.s. denotes no statistical significance.

Scotopic flash ERG measurements of a-waves (reflecting rod photoreceptor functions) at seven stimulus intensities ranging from 0.001–10.0 cd s/m^2^ revealed significantly reduced a-waves in *Pgc-1α*^*−/−*^ mice as compared to WT at stimulus intensities ranging from 0.1-10.0 cd·s/m^2^ (adj. *P* = 0.0003, 0.0008, 0.0005, <0.0001, <0.0001, respectively) (Fig. 2A–C). *Pgc-1α*^*+/−*^ mice, compared to WT, had significantly reduced a-wave amplitudes at the maximum stimulus intensity of 10.0 cd s/m^2^ (adj. *P* = 0.0281). Moreover, significant differences in a-wave amplitude were observed between *Pgc-1α*^*−/−*^ and *Pgc-1α*^*+/−*^ mice at all intensities in the range of 0.1–10.0 cd·s/m^2^ (adj. *P* = 0.0184, 0.0152, 0.0186, 0.0054, 0.03, respectively) (Fig. 2B). Analyses of a-wave implicit times revealed no significant differences (Fig. 2F).

Analyses of b-waves (a response primarily contributed by the bipolar-ON cells) revealed reduced b-wave amplitudes in *Pgc-1α*^*−/−*^ as compared to WT mice at all intensities in the range of 0.01-10.0 cd·s/m^2^ (adj. *P* = 0.0019, <0.0001, <0.0001, <0.0001, <0.0001, <0.0001, respectively). *Pgc-1α*^*+/−*^ mice, at the stimulus intensities of 5.0–10.0 cd·s/m^2^ had significantly reduced b-wave amplitudes compared to WT controls (adj. *P* = 0.0336, 0.0136, respectively) (Fig. 2A, D, E). In addition, the b-wave amplitudes of *Pgc-1α*^*−/−*^ were reduced compared to *Pgc-1α*^*+/−*^ mice at all intensities ranging from 0.1–10.0 cd s/m^2^ (adj. *P* = 0.0189, 0.0101, 0.0153, 0.0125, 0.0451, respectively) (Fig. 2D). No differences in b*-wave* implicit times were observed across the three groups (Fig. 2F).

### Inhibition of *PGC1A* reduced the expression of PPARs and LXRs, declined fatty acid β-oxidation, and increased lipid peroxidation in human RPE cells

**Fig. 3:**
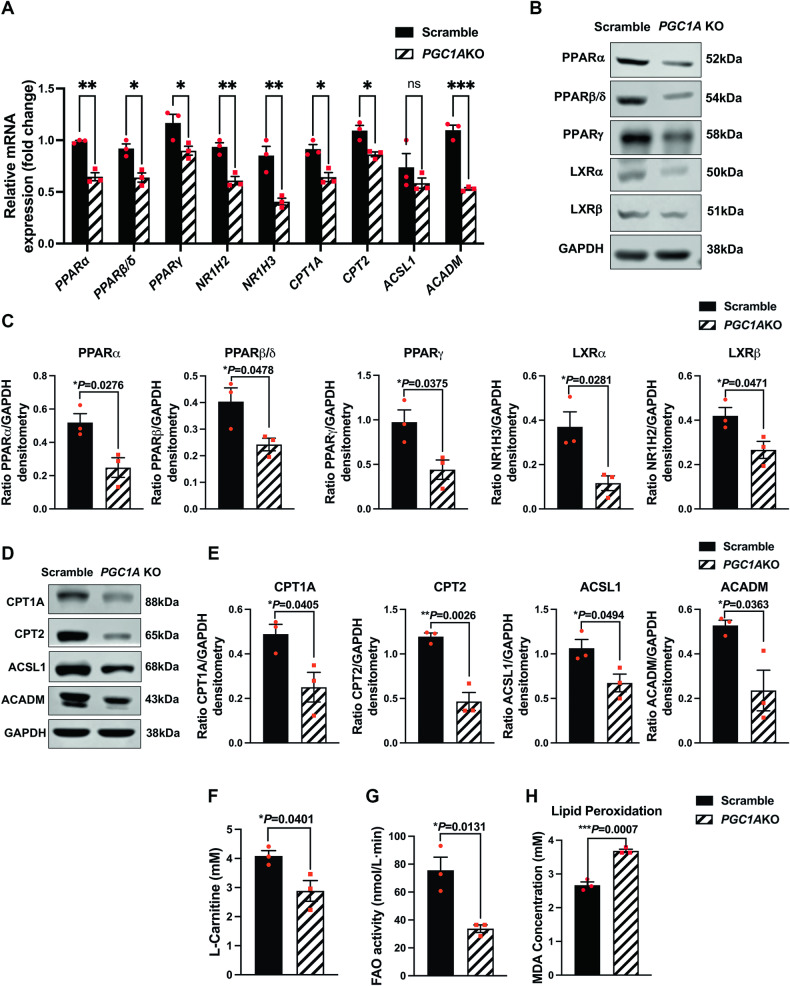
Inhibition of *PGC1A* reduced the expression of PPARs and LXRs, decreased fatty acid β-oxidation, and increased lipid peroxidation in human RPE cells. **A** qPCR was performed to quantify mRNA levels of *PPARα, PPARβ, PPARγ, NR1H3 & NR1H2, CPT1A, CPT2, ACSL1*, and *ACADM* (*n* = 3) in ARPE19-*PGC1A* KO and control cell lines. **B**, **C** Western blot of protein levels of PPARα, PPARβ, PPARγ, LXRα, and LXRβ. **D**, **E** Western blot of protein levels of CPT1A, CPT2, ACSL1, and ACADM. GAPDH was used for internal control and statistical normalization (*n* = 3). **F** L-Carnitine content was significantly reduced in ARPE-*PGC1A* KO cells compared to that in the Scramble control (*n* = 3). **G** FAO levels were significantly reduced following *PGC1A* inhibition in ARPE19 cells (*n* = 3). **H** Higher MDA concentration, representing increased lipid peroxidation levels, was observed in ARPE-*PGC1A* KO compared to control cells. ImageJ was used for densitometry analysis. Unpaired *t*-test was performed for statistical analysis. Graph represents mean ± SEM, *P ≤ 0.05, **P ≤ 0.01, ***P ≤ 0.001, n.s. means no significance.

PGC-1α functions as a regulator of lipid metabolic pathways [10] by influencing various transcription factors and nuclear hormone receptors, including *PPARα, PPARβ, PPARγ, NR1H3 & NR1H2* (encode *LXRα* & *LXRβ*, respectively). These transcription factors were reported to be involved in different stages of lipid metabolism such as fatty acid (FA) uptake [25], FA cytoplasmic transport [11], lipid lysosomal degradation [26], fatty acid β-oxidation (FAO) [27], and lipid biosynthesis [27]. We generated the ARPE19-*PGC1A* KO cell line mediated by CRISPR-Cas9 technology to inhibit *PGC1A* gene expression (S-Fig. 1) and to subsequently explore the mechanisms by which PGC1α regulates lipid metabolism in RPE cells. qPCR and Western blot analyses showed a significant reduction in mRNA and protein levels of *PPARα*, *PPARβ, PPARγ, NR1H3 & NR1H2* in ARPE19-*PGC1A* KO as compared to Scramble-gRNA (control) cells (Fig. 3A–C). To confirm that the data obtained by CRISPR-Cas9 inhibition of *PGC1A* in RPE are not due to off-target effects of CRISPR-Cas9, we used siRNA to inhibit *PGC1A* in ARPE19 cells. Similar results were observed when *PGC1A* was inhibited by siRNA in ARPE19 cells (S-Fig. 2A–C).

Long-chain fatty acids are converted into their active form, the acyl-CoAs, by acyl-CoA synthetase long-chain family member (ACSL) for both lipid synthesis and lipid degradation via FAO [28]. Fatty acids are transported into mitochondrial matrix by the sequential action of carnitine palmitoyltransferase I (CPT1) in the outer mitochondrial membrane and carnitine palmitoyltransferase II (CPT2) in the inner membrane, along with carnitine-acylcarnitine translocase [29]. Mitochondrial β-oxidation of long-chain fatty acids is initiated by the medium-chain specific Acyl-Coenzyme A dehydrogenase (ACADM) [29].

Our data showed a significant decrease in *CPT1A, CPT2, ACSL1,* and *ACADM* gene expression (Fig. 3A). Western blot analyses further revealed reduced protein levels of CPT1A, CPT2, ACSL1, and ACADM in ARPE19-*PGC1A* KO as compared to control cells (Fig. 3D, E). We observed similar results when we used siRNA to knock down *PGC1A* in ARPE19 cells (S-Fig. 2A–C). In addition, L-Carnitine activity was decreased in ARPE19-*PGC1A* KO cells (Fig. 3F). As CPT1A transports FAs into the mitochondria in a carnitine-dependent manner, blunted L-Carnitine activity further exacerbates FAO deficiency. The FAO assay showed a significant reduction in FAO efficiency in ARPE19-*PGC1A* KO as compared to control cells (Fig. 3G). Analysis of malondialdehyde (MDA) concentration revealed increased lipid peroxidation in ARPE19-*PGC1A* KO as compared to control cells (Fig. 3H).

### Inhibition of *PGC1A* induces lipid droplet generation in RPE cells

**Fig. 4:**
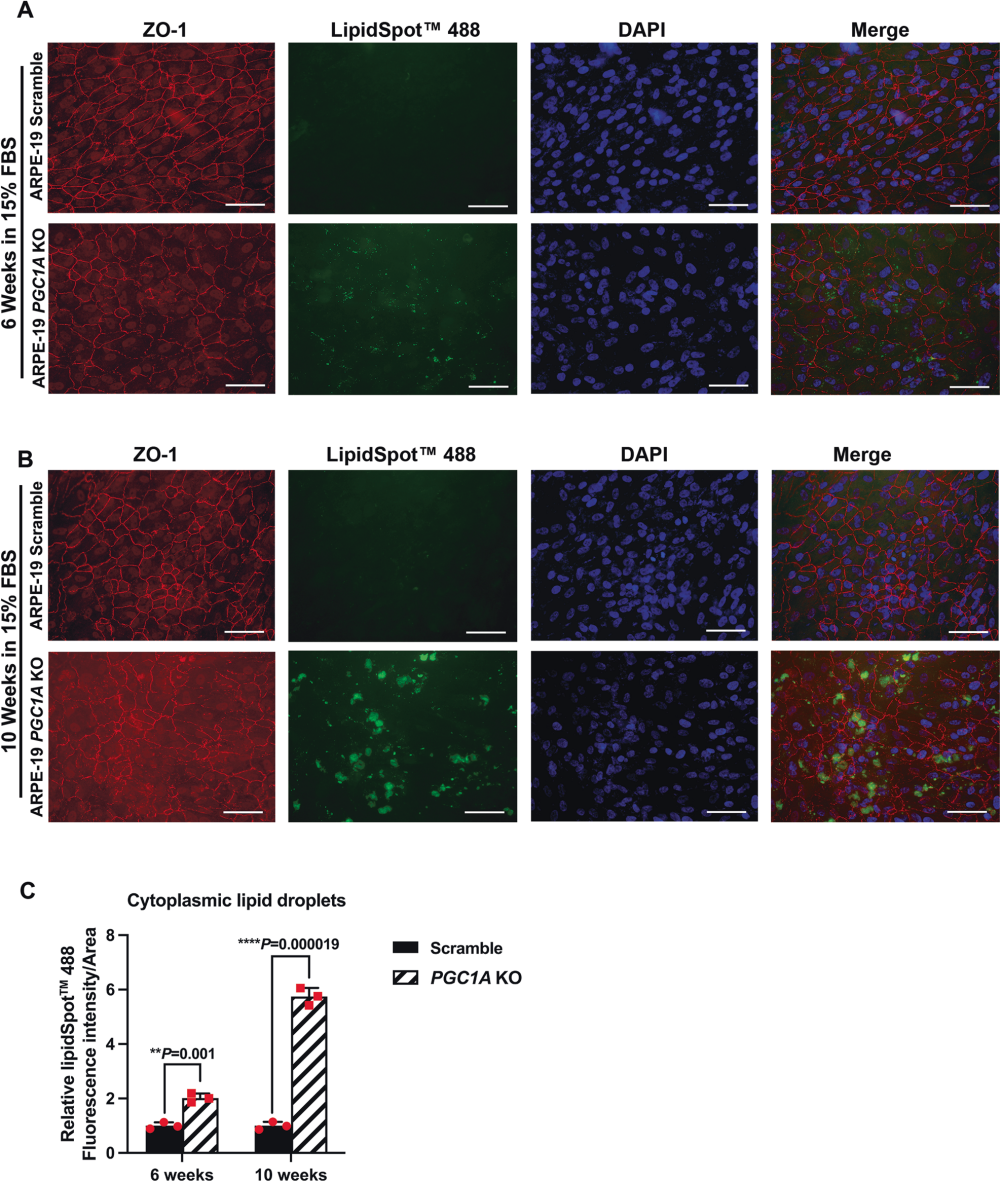
Inhibition of *PGC1A* results in lipid droplet accumulation in ARPE 19 cells. ARPE19-*PGC1A* KO cells and Scramble control cells were treated with 2 μM doxycycline for 14 days to induce the CRISPR-Cas9 gene editing process. Lipid droplets were stained by LipidSpot 488 in both ARPE19-*PGC1A* KO and Scramble control groups subjected to treatment with medium containing 15% FBS for 6 weeks (**A**) and 10 weeks (**B**), respectively. The tight junction protein ZO-1 was stained in red, and nuclei were stained in blue by DAPI. The scale bar is 100 μM. **C** Relative quantification of lipid droplets in ARPE19-Scramble and ARPE19-*PGC1A* KO cells at 6 weeks and 10 weeks post-incubation with media containing 15% FBS. Unpaired *t*-test was performed for statistical analysis. Graph represents mean ± SEM, *P ≤ 0.05, **P ≤ 0.01, *** P ≤ 0.001, ****P ≤ 0.0001, n.s. means no significance.

We cultured ARPE19-*PGC1A* KO cells and inhibited *PGC1A* expression by doxycycline treatment for 14 days. Then ARPE19-*PGC1A* KO and control cells were cultured in complete medium with 15% FBS, which supplies higher lipids, triglycerides, and cholesterol to cells and mimics a mild high-fat culturing condition. Immunostaining analyses revealed that 6 weeks post 15% FBS medium treatment multiple, small lipid droplets (green) appeared in the cytoplasm of ARPE19-*PGC1A* KO cells. Under normal culture conditions, ARPE19 cells can uptake fatty acids and process them by β-oxidation for cell energy needs. Therefore, we did not observe lipid droplet accumulation in the control group at 6 weeks post 15%FBS medium treatment (Fig. 4A, C). After 10 weeks incubation with medium containing 15% FBS, lipid droplets were enlarged, and the tight junctions (ZO-1, red) were dramatically disarranged and hexagonal RPE morphology was disrupted, showing an epithelial-to-mesenchymal transition in *PGC1A* KO cells. We also observed minor lipid droplet formation in ARPE19 Scramble cells, since long-term culture of RPE cells in the presence of 15% FBS gradually induces lipid droplets (Fig. 4B, C).

### Inhibition of *PGC1A* promotes cholesterol esterification while reducing triglyceride biosynthesis, limits uptake of exogenous LDL, decreases cholesterol efflux, and lowers the inhibition of the rate-limiting enzyme in cholesterol biosynthesis, HMGCR, in RPE cells

Lipid droplets (LDs) are organelles that store neutral lipids which are mainly composed of triglycerides (TAG) and cholesteryl esters (CE) [30]. Cells can catalyze the conversion of diacylglycerol and fatty acyl CoA to TGA by diacylglycerol O-acyltransferase 1 and 2 (DGAT1 and DGAT2) [31] and/or catalyze the formation of CE from fatty acids (FAs) and cholesterol, or retinol by sterol O-acyltransferase 1 and 2 (SOAT1 and SOAT2) [32].

**Fig. 5:**
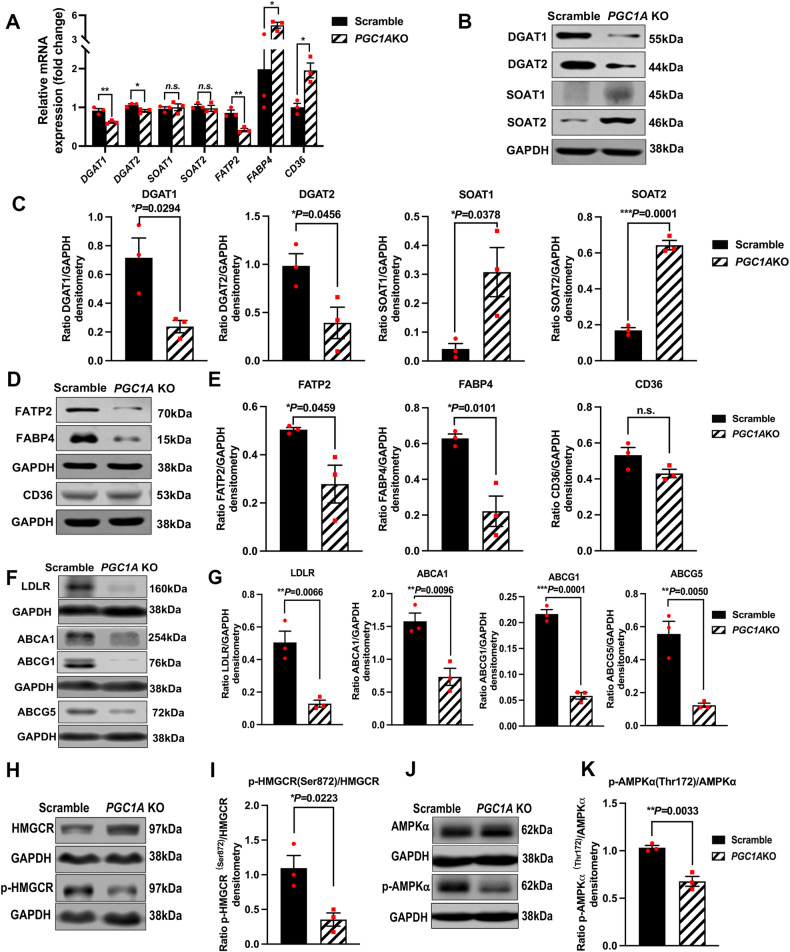
Inhibition of *PGC1A* reduces triglyceride biosynthesis while promoting cholesterol esterification and biosynthesis, affects fatty acid uptake, and decreases cholesterol efflux in the RPE. **A** qPCR analysis of mRNA levels of *DGAT1*, *DGAT2*, *SOAT1*, *SOAT2*, *FATP2, FABP4*, and *CD36* (*n* = 3). **B**, **C** Protein levels of DGAT1, DGAT2, SOAT1 and SOAT2 (*n* = 3). **D**, **E** Protein levels of FATP2, FABP4, and CD36 (*n* = 3). **F**, **G** *PGC1A* inhibition significantly reduced protein levels of low-density lipoprotein receptor (LDLR) and cholesterol efflux-related proteins, ABCA1, ABCG1, and ABCG5 (*n* = 3). **H**–**K** Inhibition of *PGC1A* significantly decreased phosphorylation of HMGCR at Ser872 and reduced phosphorylation of AMPKα at Thr172 (*n* = 3). GAPDH was used for internal control and statistical normalization (*n* = 3). ImageJ was used for densitometry analysis. Unpaired *t*-test was performed for statistical analysis. Graph represents mean ± SEM, *P ≤ 0.05, **P ≤ 0.01, *** P ≤ 0.001, n.s. means no significance.

To investigate the mechanism by which lipid droplets accumulate in ARPE19-*PGC1A* KO cells, we verified mRNA and protein levels of *DGAT1, DGAT2, SOAT1*, and *SOAT2*. Our data showed that both mRNA expression and protein levels of DGAT1 and DGAT2 were significantly decreased in ARPE19-*PGC1A* KO as compared to control cells (Fig. 5A–C). However, SOAT1 and SOAT2 protein levels were significantly increased (Fig. 5B, C), while no significant changes were observed in their mRNA levels (Fig. 5A). These findings suggest that LDs shown in Fig. 4 may dominantly result from the formation of CE rather than TAG synthesis.

Exogenous FAs and lipids (such as cholesterol and retinol) can be catalyzed into CEs which ultimately contribute to the formation of LDs, particularly in conditions of impaired fatty acid oxidation. To explore whether CEs in LDs are mainly derived from FAs or cholesterol, we first examined the expression of three fatty acid transport-related proteins, namely FATP2, FABP4, and CD36, which are closely associated with intracellular FA accumulation. FATP2, also known as SLC27A2, can mediate the uptake of exogenous FAs or lipids and convert free long-chain fatty acids into fatty acyl-CoA esters, thereby playing a key role in lipid biosynthesis and degradation [33]. Elevation of FATP2 levels in the liver results in increased fat accumulation and inflammation [34]. FATP2 abundance also contributes to excessive FA uptake and impaired FAO activities in renal tubular epithelial cells [35]. However, in our study, we observed that inhibition of *PGC1A* led to the reduction of FATP2 at both the mRNA and protein levels (Fig. 5A, D, E). Fatty acid-binding protein 4 (FABP4) is a member of the fatty acid-binding protein family and acts as an intracellular lipid chaperone to regulate lipid trafficking in cells. It has been reported that upregulation of FABP4 promotes the deposition of LDs in macrophages [36]. Here, we found that inhibition of *PGC1A* in ARPE19 cells induced a decrease in FABP4 protein levels, while mRNA levels were significantly increased (Fig. 5A, D, E). CD36 is also known as a pivotal long-chain fatty acid transporter that contributes to fatty acid accumulation [37] and negative regulation of lipophagy [38]. We found no change in the protein levels of CD36 in ARPE19-*PGC1A* KO compared to control cells, while its mRNA levels were significantly increased (Fig. 5A, D, E). These findings suggest that the CEs in LDs were not dominantly converted from exogenous FAs transported by FATP2, FABP4, and CD36.

Subsequently, we assessed the expression of LDLR, involved in the uptake of exogenous cholesterol. Surprisingly, inhibition of *PGC1A* also led to a significant reduction in LDLR expression (Fig. 5F, G). Furthermore, we measured the levels of cholesterol efflux-related proteins ABCA1/ ABCG1/ABCG5 and found that their levels were significantly decreased in ARPE19-*PGC1A* KO compared to control cells (Fig. 5F, G).

To further explore the possibility of endogenous cholesterol biosynthesis, we tested HMG-CoA reductase (HMGCR), the rate-limiting enzyme in cholesterol synthesis [39], and analyzed its phosphorylation at Ser872. In humans, decreased phosphorylation of HMGCR (p-HMGCR) at Ser872 is linked to increased HMGCR activity [40]. Our data showed that inhibition of *PGC1A* significantly decreased phosphorylation levels of HMGCR (Fig. 5H, I), suggesting that HMGCR activity is increased in ARPE19-*PGC1A* KO cells. It has been reported that AMP-activated protein kinase (AMPK) is the dominant upstream kinase involved in phosphorylation of HMGCR [41, 42]. AMPK is activated upon its phosphorylation at Thr172 and, in turn, inactivates HMGCR via phosphorylation of the enzyme at Ser872. Thus, we examined AMPKα and its phosphorylation (p-AMPKα) at Thr172, and found that inhibition of *PGC1A* led to a significant reduction in p-AMPKα levels (Fig. 5J, K). Our data indicates that *PGC1A* inhibition decreases the kinase activity of AMPK and, consequently, elevates HMGCR activity.

These findings suggest that, while the transport of exogenous LDL was decreased by *PGC1A* inhibition, cholesterol biosynthesis was induced by increased HMGCR activity in ARPE19-*PGC1A* KO cells. Additionally, reduced cholesterol efflux might contribute to the formation of cholesterol esters and, ultimately, the generation of lipid droplets.

### Inhibition of *PGC1A* in human RPE cells downregulates mitochondrial biosynthesis and activity, decreases antioxidant enzymes and mitochondrial membrane potential, and increases susceptibility to oxidative stress

PGC-1α regulates the expression of nuclear and mitochondrial genes that encode components of the electron transport system and oxidative phosphorylation (OXPHOS) machinery via the coactivation of nuclear respiratory factors 1 (NRF1) and estrogen-related receptors α, β, and γ (ERR-α, -β and -γ) [9]. These effects can further modulate the expression of mitochondrial transcription factor A (TFAM), which is known to control mitochondrial DNA (mtDNA) replication and transcription, therefore regulating cellular oxidative metabolism [9].

**Fig. 6:**
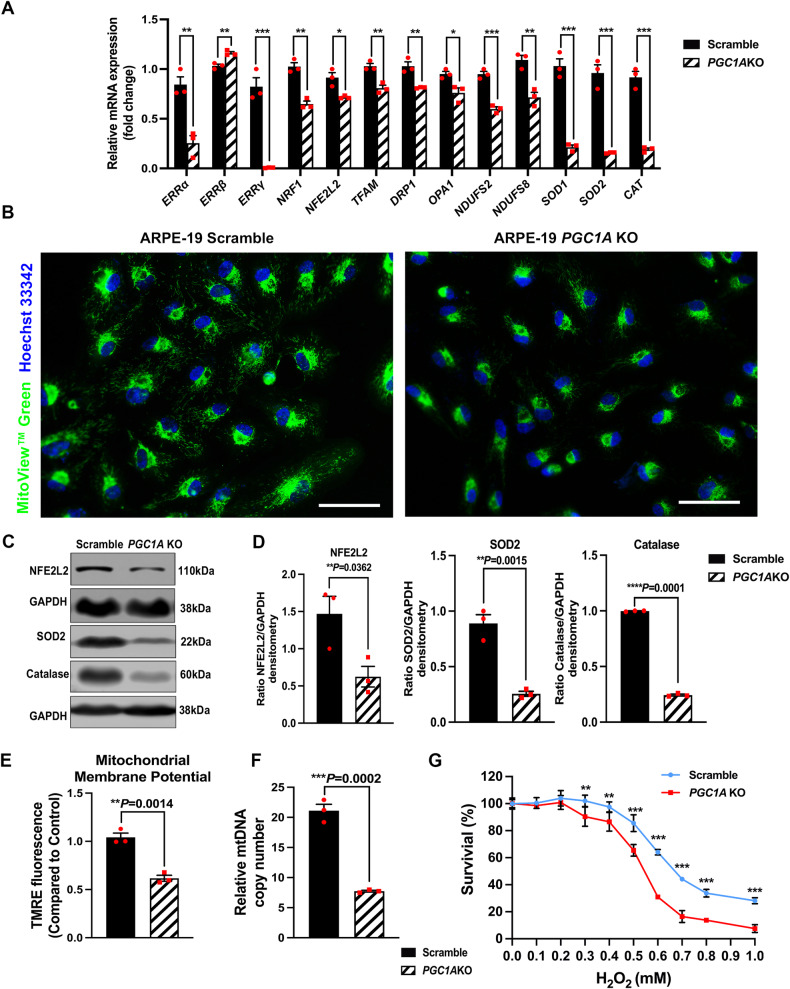
Inhibition of *PGC1A* in RPE cells reduced mitochondrial mass and activity, mitochondrial membrane potential, antioxidant enzymes, and increased susceptibility to oxidative stress. **A** mRNA levels of mitochondrial biosynthesis-related transcription factors *ERRα, ERRβ, ERRγ, NRF1*, and *TFAM*; mitochondrial dynamics genes, *DRP1* and *OPA1*; mitochondrial respiratory chain complex1 core subunit genes, *NDUFS2* and *NDUFS8*; oxidative stress-regulating gene, *NFE2L2;* and antioxidant enzyme genes *SOD1, SOD2*, and *CAT* (*n* = 3). **B** Immunostaining of mitochondria (green) by MitoView, showing reduced mitochondrial mass in ARPE19-*PGC1A* KO compared to Scramble control cells. Hoechst 33342 (blue) was used to stain nuclei. The scale bar represents 100μm. **C**, **D** Protein levels of NFE2L2, SOD2, and Catalase were significantly decreased in ARPE19-*PGC1A* KO cells (*n* = 3). **E** Mitochondrial membrane potential quantification in ARPE19-*PGC1A* KO and Scramble control groups (*n* = 3). **F** mtDNA copy number was significantly reduced in ARPE19-*PGC1A* KO as compared to Scramble control cells (*n* = 3). **G** Cell viability was significantly lower in ARPE19-*PGC1A* KO cells as compared to the control group under increasing concentration of H_2_O_2_ treatment for 24 h (*n* = 3). GAPDH was used for internal control and statistical normalization (*n* = 3). ImageJ was used for densitometry analysis. Unpaired *t*-test was performed for statistical analysis. Graph represents Mean ± SEM, *P ≤ 0.05, **P ≤ 0.01, ***P ≤ 0.001, ****P ≤ 0.0001.

qPCR analyses showed a significant decrease in mRNA levels of *ERRα, ERRγ, NRF1* and *TFAM* in ARPE19-*PGC1A* KO compared to control cells, whereas *ERRβ* mRNA levels were significantly increased (Fig. 6A). We inhibited *PGC1A* with siRNA and obtained similar results in ARPE19 cells (S-Fig. 2A, B). Furthermore, mitochondrial DNA (mtDNA) copy number was reduced in ARPE19-*PGC1A* KO cells (Fig. 6F). MitoView staining revealed a significant reduction of mitochondrial mass consistent with reduced mtDNA copy number in ARPE19-*PGC1A* KO (Fig. 6B).

Analyses of mitochondrial dynamics-related genes, *DRP1 and OPA1*, and mitochondrial respiratory chain complex1 core subunits genes, *NDUFS2* and *NDUFS8*, confirmed a reduction in their mRNA levels in ARPE19-*PGC1A* KO compared to control cells (Fig. 6A). Measurement of mitochondrial membrane potential revealed a significant decrease in ARPE19-*PGC1A* KO compared to control cells (Fig. 6E).

Previous studies have confirmed that PGC1α interacts with nuclear factor E2-related factor 2 (NFE2L2), regulating its expression and activity [43, 44]. NFE2L2 can regulate the antioxidant response to protect cells against oxidative damage by inducing the production of antioxidant enzymes, including superoxide dismutase 1/2 (SOD1/2) and catalase (CAT) [45, 46]. In our current research, we found that inhibition of *PGC1A* downregulated mRNA levels of *NFE2L2, SOD1, SOD2, and CAT* (Fig. 6A) and reduced protein levels of NFE2L2, SOD2, and Catalase (Fig. 6C, D). We did not observe a reduction in the nuclear localization of NFE2L2 (S-Fig. 3) in ARPE19-*PGC1A* KO cells, suggesting that the downregulation of *SOD1, SOD2*, and *CAT* mRNA levels originated from decreased NFE2L2 protein levels.

Our cell viability analyses of ARPE19-*PGC1A* KO under increasing concentrations of H_2_O_2_ revealed increased susceptibility to oxidative stress compared to control cells at 24 h

of H_2_O_2_ incubation (Fig. 6G). Similar results were obtained when we used siRNA to inhibit *PGC1A* in ARPE19 cells (S-Fig. 2D).

### Inhibition of *PGC1A* induces loss of cardiolipin in human RPE cells

Cardiolipin (CL) is a unique dimeric glycerophospholipid localized almost exclusively to the mitochondria in mammalian cells [47], and plays a critical role in the homeostasis of the mitochondrial membrane, thus influencing mitochondrial OXPHOS and FAO.

**Fig. 7:**
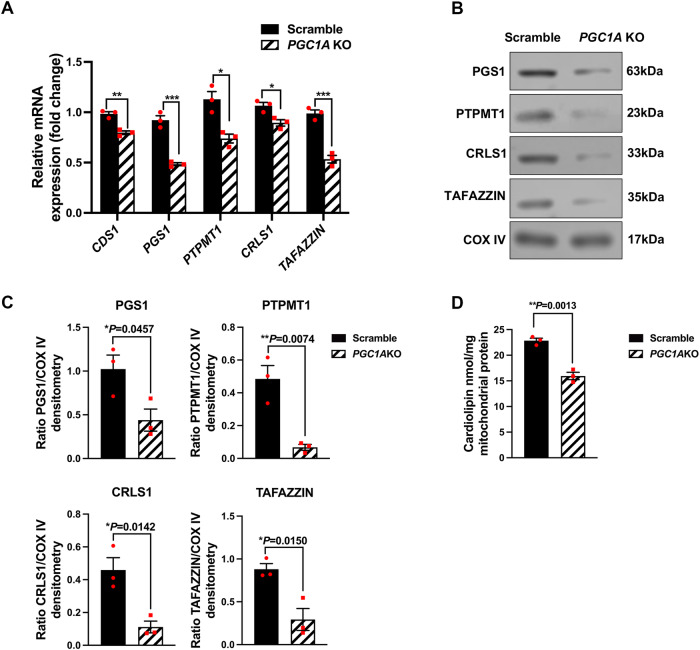
*PGC1A* deficiency in ARPE19 cells downregulates cardiolipin synthesis. **A** mRNA levels of cardiolipin synthesis pathway-related genes *CDS1, PGS1, PTPMT1, CRLS, and TAFAZZIN* (*n* = 3). **B**, **C** Western blot analysis of PGS1, PTPMT1, CRLS1, and TAFAZZIN protein levels (*n* = 3). ImageJ was used for densitometry analysis. **D** *PGC1A* inhibition in ARPE19 cells significantly decreased cardiolipin content compared to control cells (*n* = 3). ImageJ was used for densitometry analysis. Unpaired *t*-test was performed for statistical analysis. Graph represents mean ± SEM, *P ≤ 0.05, **P ≤ 0.01, ***P ≤ 0.001.

To elucidate the mechanism by which *PGC1A* inhibition affects cardiolipin synthesis, we performed qPCR to measure mRNA levels of genes involved in cardiolipin synthesis pathway, *CDS1*, *PGS1*, *PTPMT1*, *CRLS1*, and *TAFAZZIN*, and found their expression to be downregulated in ARPE19-*PGC1A* KO cells (Fig. 7A). Our Western blot data showed that protein levels of PGS1, PTPMT1, CRLS1, and TAFAZZIN were significantly reduced (Fig. 7B,C). Furthermore, we applied equal amounts of mitochondrial protein from the ARPE19-*PGC1A* KO and the control groups to test the mitochondrial cardiolipin content, and we found that inhibition of *PGC1A* significantly decreased cardiolipin levels (Fig. 7D). These data suggest that the loss of cardiolipin in ARPE19-*PGC1A* KO cells resulted from the inhibition of cardiolipin synthesis rather than reduced mitochondrial mass as shown in Fig. 6B.

## Discussion

Our study reports on essential roles for PGC-1α in RPE metabolic homeostasis, lipid droplet formation, mitochondrial functions, and cellular resistance to oxidative stress. Hallmark phenotypic and functional changes associated with PGC-1α downregulation in mice include RPE, retinal dysfunction, and lipid deposits as observed by ERG and funduscopy, respectively. The inhibition of *PGC1A* in vitro in the RPE induced dysfunction of lipid metabolism and FA β-oxidation, accumulation of lipid droplets, reduction of triglyceride biosynthesis, and induction of cholesteryl ester conversion. *PGC1A* deficiency reduced the exogenous uptake of LDL and decreased cholesterol efflux while enhancing the activation of HMGCR, the rate-limiting enzyme in cholesterol biosynthesis. *PGC1A* inhibition also resulted in the downregulation of mitochondrial biosynthesis, dynamics, and activity-related genes, reduction of mitochondrial membrane potential and antioxidant enzymes, and elevation of RPE susceptibility to oxidative stress and cell death via lipid peroxidation. Collectively, our study reveals that inhibition of PGC-1α contributes to the formation of AMD-like phenotypes in the RPE and induces retinal degeneration by dysregulating lipid metabolism.

### RPE and retinal degeneration in *Pgc-1α* knockout and heterozygous mice

A growing body of evidence suggests that PGC-1α might have important implications in RPE and retinal function [17–20]. However, the mechanisms by which PGC-1α regulates RPE and retinal function remain to be elucidated.

We have previously shown that *Pgc-1α* repression combined with a high-fat diet can induce drastic AMD-like phenotypes in mice [21]. Since PGC-1α is identified as a key regulator of lipid and metabolic regulation [10], a combination of a high-fat diet and *Pgc-1α* repression can accelerate and exacerbate the formation of disease phenotypes in mice.

Here, we show that inhibition of *Pgc-1α* alone can also affect RPE and retinal function.

We analyzed the RPE and retinal health and function in *Pgc-1α*^*+/−*^ and *Pgc-1α*^*−/−*^ mice using fundus photography and ERG. Our fundus imaging showed bright spots in the RPE of *Pgc-1α*^*+/−*^ and *Pgc-1α*^*−/−*^ mice resembling drusen deposits in humans. The ERG confirmed retinal degeneration, shown by a significant reduction in a- and b-waves, and RPE dysfunction, shown by a significant decrease in c-waves in *Pgc-1α*^*+/−*^ and *Pgc-1α*^*−/−*^ mice. A recent study on the RPE-specific PGC-1α inhibition in mice showed a decrease in a-, b- and c-waves [20]. Here, we show RPE and retinal degeneration in *Pgc-1α*^*+/−*^ mice, which indicates that partial inhibition of *Pgc-1α* can also induce RPE and retinal degeneration over time. These observations are physiologically more relevant to human conditions where PGC-1α expression could be declined by aging [48, 49].

### Lipid droplet generation and lipid metabolism in RPE

Drusen, formed between the RPE and Bruch’s membrane, are a distinctive feature of dry AMD and contribute to RPE and photoreceptor degeneration [23]. A recent study demonstrated impaired cholesterol efflux in the RPE of individuals with juvenile macular degeneration [50]. However, the mechanisms underlying these cellular dysfunctions remain to be elucidated.

To investigate the role of PGC-1α in lipid metabolism in RPE, we used CRISPR/Cas9 gene editing to inhibit *PGC1A* expression in ARPE19 cells. ARPE19-*PGC1A* KO cells showed elevated lipid droplet generation, which increased in size with time in culture. These observations indicate a direct role for PGC-1α in regulating lipid droplet formation in the RPE and suggest that the pharmacological activation of PGC-1α might be beneficial in reducing lipid and drusen formation in AMD patients. These data also support a role for PGC-1α in AMD, as we have previously reported lipid droplet accumulation [6] and dysregulated AMPK-SIRT1-PGC-1α and metabolic pathways [22] in the RPE derived from AMD donor eyes [23].

To delineate the mechanism by which PGC-1α regulates lipid droplet generation in RPE, we measured the expression of genes and proteins responsible for TAG synthesis and cholesterol esterification. Our data showed that increased lipid droplet formation under *PGC-1A* inhibition was not due to the synthesis of TAG but rather derived from cholesterol esterification catalyzed by SOAT1 and SOAT2.

*PGC1A* inhibition also impaired the regular uptake of exogenous FAs mediated by FATP2 and FABP4 and limited the exogenous LDL uptake since we observed lower LDLR protein levels in *PGC1A KO* cells. However, *PGC1A* deficiency enhanced the activation of HMGCR via a decrease of p-HMGCR at Ser872 achieved by downregulating p-AMPK at Thr172, suggesting elevated cholesterol biosynthesis. Moreover, our data demonstrated decreased ABCA1/ABCG1/ABCG5 protein levels, indicating reduced cholesterol efflux. These results suggest that lipid droplet generation in ARPE19-*PGC1A* KO cells is likely due to dysfunctional FAO, excessive cholesterol biosynthesis and esterification, and deficient cholesterol efflux.

### Mitochondrial biosynthesis and activity, oxidative stress, and mitochondrial membrane homeostasis in RPE

PGC-1α, a crucial regulator of mitochondrial biogenesis, plays a pivotal role in increasing mitochondrial mass and activity [9]. Previously, we showed reduced mitochondrial Complex I activity and mtDNA copy number in Pgc-1α^+/−^ mice [21]. Another study showed that PGC-1α silencing induced mitochondrial dysfunction and oxidative stress in RPE [20].

To further investigate the role of PGC-1α in mitochondrial activity and integrity, we measured the expression of mitochondrial activity- and dynamic-related genes and mtDNA copy number, all of which were declined in ARPE19-*PGC1A* KO as compared to control cells. Moreover, mitochondrial mass and membrane potential were significantly reduced in ARPE19-*PGC1A* KO compared to control cells. The antioxidant enzymes SOD2 and Catalase were also repressed in ARPE19-*PGC1A* KO cells due to reduced expression of NFE2L2, which regulates the transcription of *SOD2* and *CAT*.

Analysis of cell viability revealed increased susceptibility to oxidative stress-induced cell death in ARPE19-*PGC1A* KO, possibly due to increased lipid peroxidation and oxidative stress.

CL, a diphosphatidylglycerol, is exclusively synthetized and primarily localized in the mitochondria [15, 47]. It plays a significant role in mitochondrial membrane homeostasis and influences OXPHOS and FAO^12^. Our investigation into the role of PGC-1α in CL regulation has yielded intriguing results. We discovered that CL content decreases upon *PGC1A* inhibition, significantly reducing the expression of genes and proteins responsible for CL biosynthesis. These findings highlight the essential role of PGC-1α in mitochondrial membrane homeostasis, activity, and dynamics and open up exciting avenues for future research in this field.

**Fig. 8:**
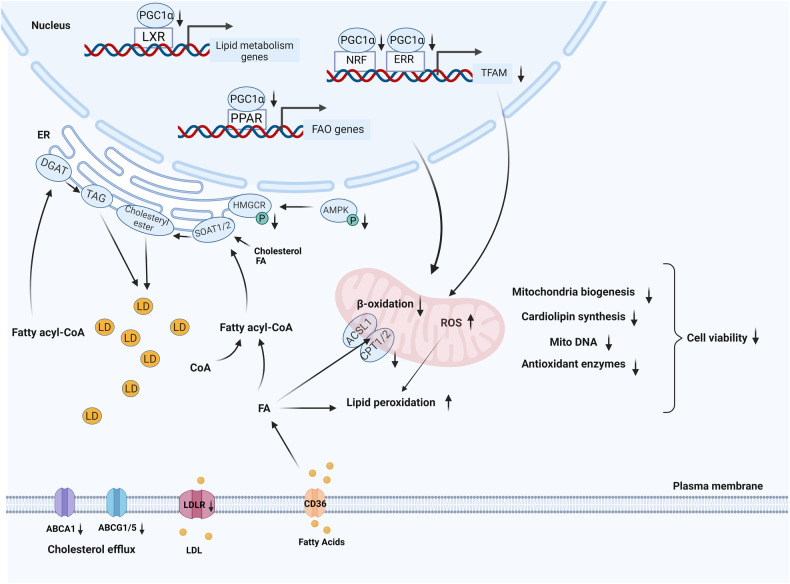
The proposed model of lipid metabolism dysregulation caused by *PGC1A* deficiency in the RPE. *PGC1A* deficiency downregulated lipid metabolism-related transcription factors such as PPARs and LXRs, reduced fatty acid β-oxidation (FAO), affected active fatty acid (FA) transportation and low-density lipoprotein (LDL) uptake, and decreased cholesterol efflux. Furthermore, *PGC1A* repression may reduce triglyceride biosynthesis by downregulating the expression of DGAT1/2 while promoting cholesterol esterification by increasing SOAT1/2 protein levels. Inhibition of *PGC1A* decreased the inhibitory phosphorylation of HMGCR by AMPK, increasing HMGCR activity and promoting cholesterol synthesis. These changes ultimately contributed to the accumulation of lipid droplets. In addition, the repression of *PGC1A* led to reduced mitochondrial mass, lower mitochondrial membrane potential, and decreased cardiolipin content, further hindering FAO. Moreover, accumulation of lipids or fatty acids in the cytoplasm increased lipid peroxidation, especially under elevated levels of reactive oxygen species (ROS), resulting from deficiencies in antioxidant enzymes. As a result, dysregulated lipid metabolism and mitochondrial dysfunction caused by *PGC1A* inhibition can lead to RPE cell death and degeneration.

Collectively, our data reveal essential functions for PGC-1α in lipid metabolism, lipid droplet formation, mitochondrial activity, maintenance of mitochondrial homeostasis, and lipid peroxidation in the RPE. Our findings suggest that reduction of PGC-1α activity during aging could contribute to RPE senescence, retinal degeneration, and consequently, AMD pathophysiology. Our observations open new avenues for developing targeted treatment strategies for AMD. Figure 8 recapitulates the role of PGC-1α in lipid metabolism in the RPE.

### Supplementary information

Supplementary Materials

Uncropped Western Blots

## Data Availability

All data generated in this study are included in the article and the Supplementary files. Additional data can be shared by the corresponding author upon reasonable request.
