# Supplementary material for: PGC-1α repression dysregulates lipid metabolism and induces lipid droplet accumulation in the retinal pigment epithelium
Source: Cell Death Dis. 2024 Jun 1;15(6):385. doi: 10.1038/s41419-024-06762-y (PMC11144268; doi:10.1038/s41419-024-06762-y)
Supplement: Supplementary file 1 — Supplementary Materials [file 41419_2024_6762_MOESM1_ESM.pdf]

Supplementary Figure 1

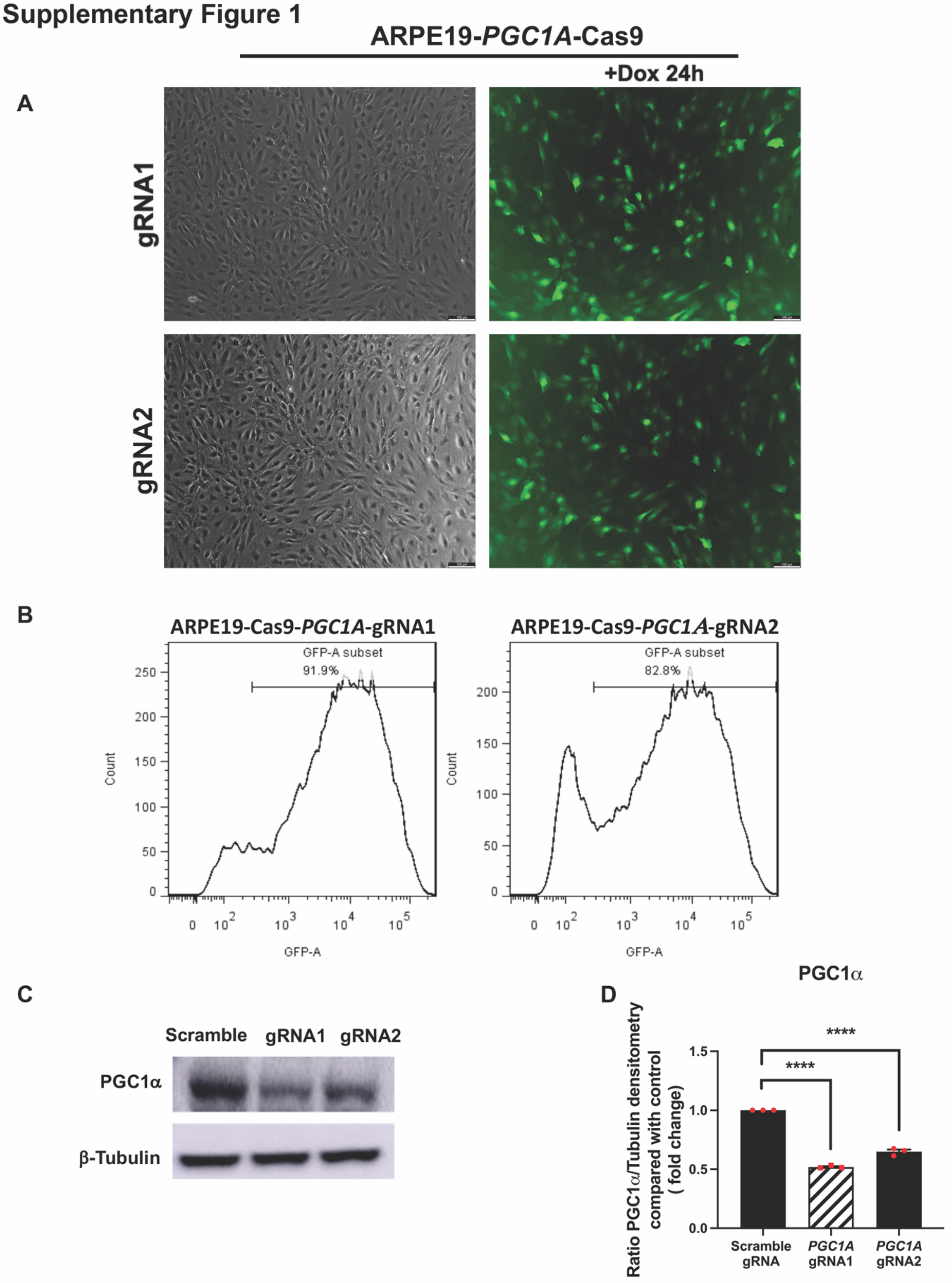

S-Figure-1. Identification of ARPE19-*PGC1A* KO cells using CRISPR/Cas9 gene editing.

ARPE19 cells were transfected with CRISPR/Cas9-*PGC1A* sgRNAs vectors and selected by 2μg/ml puromycin for 2 weeks. (**A**) ARPE19-*PGC1A* KO cells were incubated with 2μM Doxycycline (DOX) for 24h to test the eGFP to confirm the expression of Cas9 (Scale bars:100μm). (**B**) Flow cytometry was performed to analyze the ratio of Cas9-eGFP cells upon 2μM doxycycline treatment for 48h. (**C-D**) PGC1α protein levels in Scramble control and 2 different sgRNA groups were analyzed by Western Blots. PGC1α protein was inhibited ~ 49% by sgRNA1 and 35% by sgRNA2 as compared with the control group. We chose gRNA1 for all of our experiments. The densitometry analysis was completed using ImageJ software. Ordinary one-way ANOVA with Dunnett's multiple comparisons were performed using GraphPad Prism 8.3.0. Graph represents mean±SD, n=3, ****$P < 0.0001$.

Supplementary Figure2

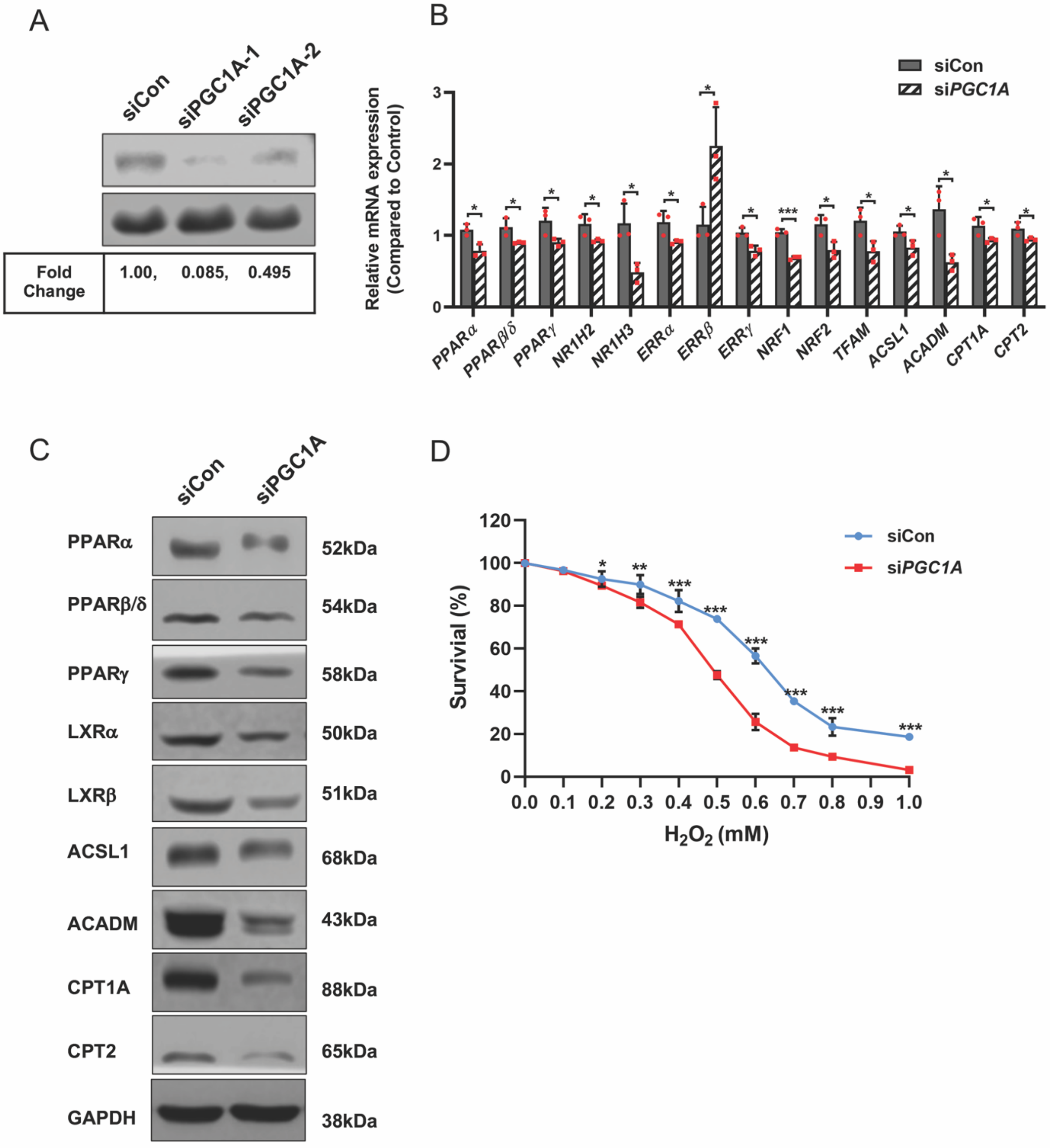

S-Figure-2. Inhibition of *PGC1A* by siRNA reduced the expression of PPARs, LXRs and fatty acid β–oxidation related components, decreased the expression of mitochondrial biosynthesis related genes, and increased the susceptibility to oxidative stress.

ARPE19 cells were transfected with 2 different siRNAs, respectively, and the mRNA and protein were extracted in 72h to perform qPCR and Western blots. (**A**) PGC1α protein was inhibited ~ 90% by siRNA1 and ~50% by siRNA2 as compared with the control group. We chose siRNA1 for the following experiments. (**B**) The mRNA levels of *PPARα, PPARβ, PPARγ, NR1H3* & *NR1H2; ERRα, ERRβ, ERRγ, NRF1, NFE2L2*, *TFAM; ACSL1, ACADM, CPT1A* and *CPT2* (n=3) in ARPE19-si*PGC1A* and control cell lines. (**C**) The protein levels of the PPARs, LXRs, and fatty acid β-oxidation-related components. (**D**) Inhibition of *PGC1A* by siRNA1 declined ARPE19 cell viability with increasing concentration of H_2O_2 treatment for 24h, (n=3). An unpaired *t*-test was performed using GraphPad Prism 8.3.0. Graph represents mean ± SEM, *$P \leq 0.05$, **$P \leq 0.01$, *** $P \leq 0.001$.

S-Figure3

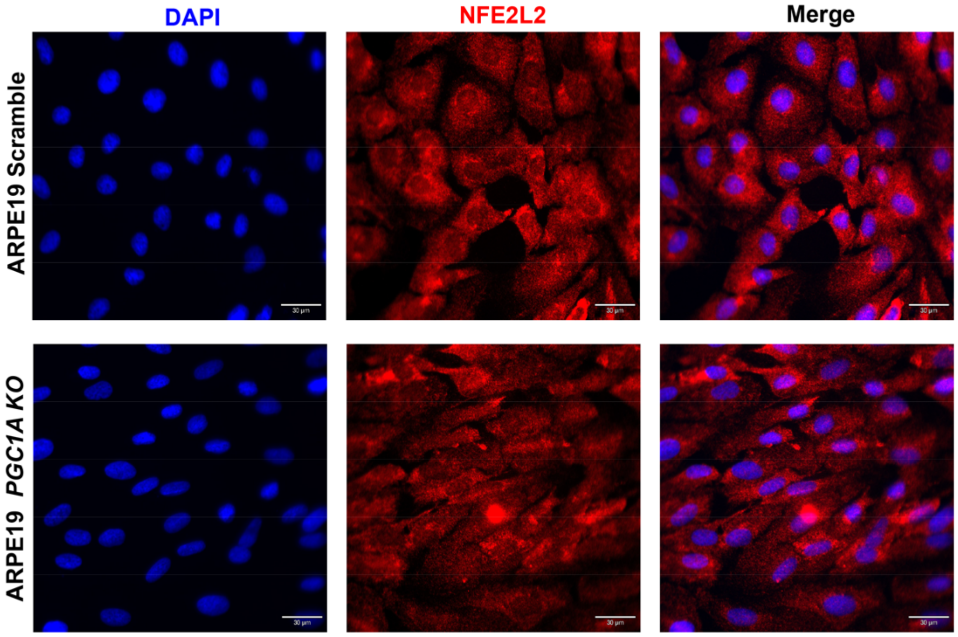

S-Figure-3. Inhibiting *PGC1A* does not affect the nuclear localization of NFE2L2 in ARPE19 cells.

Immunofluorescence images of NFE2L2 in ARPE19 PGC1A KO and the Scramble control cells.

Cells were seeded in a 35mm glass bottom confocal dish for 24h, and fixed in 4% formaldehyde for 15min at room temperature, then permeabilized with 0.25% Triton™ X-100 for 10 minutes. Cells were blocked with 5% BSA for 1hr at room temperature, followed by 3 washes with PBS, and overnight incubation with the primary antibody at 4℃. Cells were incubated with the secondary antibody the next day for 1hr at room temperature and mounted for imaging. The corresponding primary and secondary antibodies with their respective dilution factors are shown in Table. S2. NFE2L2 is stained in red (AlexaFluor 594), and nuclei were counterstained with DAPI (blue, 0100-20; Southern Biotech). Scale bars: 30 µm.

Table.S1. Primers used for qPCR analyses.

Primer Name	Primer Sequence (5'-3')
human *PPAR*α FOR<br>human *PPAR*α REV	TTCGCAATCCATCGGCGAG<br>CCACAGGATAAGTCACCGAGG
human *PPAR*β/δ FOR<br>human *PPAR*β/δ REV	CAGGGCTGACTGCAAACGA<br>CTGCCACAATGTCTCGATGTC
human *PPAR*γ FOR<br>human *PPAR*γ REV	TACTGTCGGTTTCAGAAATGCC<br>GTCAGCGGACTCTGGATTCAG
human *NR1H2* FOR<br>human *NR1H2* REV	AGAACTAATGATCCAGCAGTTGG<br>TTGCTTAGCGAAGTCCACGAT
human *NR1H3* FOR<br>human *NR1H3* REV	CCTTCAGAACCCACAGAGATCC<br>ACGCTGCATAGCTCGTTCC
human *CPT1A* FOR<br>human *CPT1A* REV	ATCAATCGGACTCTGGAAACGG<br>TCAGGGAGTAGCGCATGGT
human *CPT2* FOR<br>human *CPT2* REV	CATACAAGCTACATTTCGGGACC<br>AGCCCGGAGTGTCTTCAGAA
human *ACSL1* FOR<br>human *ACSL1* REV	CTTATGGGCTTCGGAGCTTTT<br>CAAGTAGTGCGGATCTTCGTG
human *ACADM* FOR<br>human *ACADM* REV	ACAGGGGTTCAGACTGCTATT<br>TCCTCCGTTGGTTATCCACAT
human *DGAT1* FOR<br>human *DGAT1* REV	CAATCTGACCTACCGCGATCT<br>TCGATGATGCGTGAGTAGTCC
human *DGAT2* FOR<br>human *DGAT2* REV	GAATGGGAGTGGCAATGCTAT<br>CCTCGAAGATCACCTGCTTGT
human *SOAT1* FOR<br>human *SOAT1* REV	CAAGGCGCTCTCTCTTAGATG<br>GGTCCAAACAACGGTAGGAAA

Primer Name	Primer Sequence (5'-3')
human *SOAT2* FOR<br>human *SOAT2* REV	ATGGAAACACTGAGACGCACA<br>GGTAGGATTGTATAGCCTCCCG
human *FATP2* FOR<br>human *FATP2* REV	GGCGCTCCTTATGGGTAACG<br>CTTGGCAGTATCTCTTCGACAG
human *FABP4* FOR<br>human *FABP4* REV	ACTGGGCCAGGAATTTGACG<br>CTCGTGGAAGTGACGCCTT
human *CD36* FOR<br>human *CD36* REV	AAGCCAGGTATTGCAGTTCTTT<br>GCATTTGCTGATGTCTAGCACA
human *ERR*α FOR<br>human *ERR*α REV	AGGGTTCCTCGGAGACAGAG<br>TCACAGGATGCCACACCATAG
human *ERR*β FOR<br>human *ERR*β REV	TGGAGGCCGTCAGAAATACAA<br>CAATGGCTTTTTAGCAGGTGGA
human *ERR*γ FOR<br>human *ERR*γ REV	GCCCTCACTACACTGTGTGAC<br>CCTGCTAATTTGGACTGGTCTT
human *NRF1* FOR<br>human *NRF1* REV	GCTGATGAAGACTCGCCTTCT<br>TACATGAGGCCGTTTCCGTTT
human *NFE2L2* FOR<br>human *NFE2L2* REV	TCCAGTCAGAAACCAGTGGAT<br>GAATGTCTGCGCCAAAAGCTG
human *TFAM* FOR<br>human *TFAM* REV	ATGGCGTTTCTCCGAAGCAT<br>TCCGCCCTATAAGCATCTTGA
human *ND1* FOR<br>human *ND1* REV	CCTAGCCGTTTACTCAATCCT<br>TGATGGCTAGGGTGACTTCAT
human *ACTB* FOR<br>human *ACTB* REV	CATCTCTTGCTCGAAGTCCA<br>ATCATGTTTGAGACCTTCAACA
human *GAPDH* FOR<br>human *GAPDH* REV	GGAGCGAGATCCCTCCAAAAT<br>GGCTGTTGTCATACTTCTCATGG

Primer Name	Primer Sequence (5'-3')
human *DRP1* FOR<br>human *DRP1* REV	TTTGACACTTGTGGATTTGCCA<br>AGTGACAGCGAGGATAATGGA
human *OPA1* FOR<br>human *OPA1* REV	TGTGAGGTCTGCCAGTCTTTA<br>TGTCCTTAATTGGGGTCGTTG
human *NDUFS2* FOR<br>human *NDUFS2* REV	GCTGTTATGTACCCAAGCAAAGA<br>TCCCCACTCAATTCCATCACT
human *NDUFS8* FOR<br>human *NDUFS8* REV	CCATCAACTACCCGTTCGAGA<br>CCGCAGTAGATGCACTTGG
human *CDS1* FOR<br>human *CDS1* REV	AGTTCCTCATTCGCTACCATAGA<br>GGTGTGACTGAGTGACAGTTATC
human *PGS1* FOR<br>human *PGS1* REV	TCGTGATGGCATCCCTCTAC<br>CCCCGCGTGAAGTCTAAGA
human *PTPMT1* FOR<br>human *PTPMT1* REV	CAGAGGAGGCTGTAAGAGCCA<br>TGTGGATGTATGACCGGATCT
human *CRLS1* FOR<br>human *CRLS1* REV	AGCAGTCCAGTTAATCTTGGTG<br>AGTCTTCCGGCCATAATGATAGT
human *TAFFAZIN* FOR<br>human *TAFFAZIN* REV	CACCGTGTCCAATCACCAGTC<br>TCCAACGCATCAACTTCAGGT
human *SOD1* FOR<br>human *SOD1* REV	AGGGCATCATCAATTTCGAGC<br>GCCCACCGTGTTTTCTGGA
human *SOD2* FOR<br>human *SOD2* REV	CAGACCTGCCTTACGACTATGG<br>CGTTCAGGTTGTTCACGTAGG
human *CAT* FOR<br>human *CAT* REV	ACTTTGAGGTCACACATGACATT<br>CTGAACCCGATTCTCCAGCA

Table. S2. Antibodies used for Western blots and Immunofluorescence.

Antibody	Source	Vendor	Cat. No	dilution
PGC1α	Rabbit	Millipore Sigma, USA	AB3242	1:1000
ACADM	Rabbit	Proteintech Group, USA	55210-1-AP	1:1000
CPT1A	Rabbit	Proteintech Group, USA	15184-1-AP	1:1000
ACSL1	Rabbit	Proteintech Group, USA	13989-1-AP	1:1000
PPARα	Rabbit	Proteintech Group, USA	15540-1-AP	1:600
PPARβ/δ	Mouse	Proteintech Group, USA	60193-1-Ig	1:1000
NR1H3	Rabbit	Proteintech Group, USA	14351-1-AP	1:2000
NR1H2	Mouse	Proteintech Group, USA	60345-1-Ig	1:800
FABP4	Rabbit	Proteintech Group, USA	12802-1-AP	1:5000
SOAT2	Rabbit	Proteintech Group, USA	21852-1-AP	1:1000
SOAT1	Rabbit	Cell signal technology, USA	35695	1:1000
CPT2	Rabbit	Proteintech Group, USA	26555-1-AP	1:1000
DGAT1	Rabbit	Proteintech Group, USA	11561-1-AP	1:1000
DGAT2	Rabbit	Proteintech Group, USA	17100-1-AP	1:1000
PTPMT1	Rabbit	Proteintech Group, USA	11493-1-AP	1:300
FATP2	Rabbit	Proteintech Group, USA	14048-1-AP	1:2000
CD36	Rabbit	Proteintech Group, USA	18836-1-AP	1:1000
PPARγ	Rabbit	Proteintech Group, USA	16643-1-AP	1:2000
PGS1	Rabbit	Proteintech Group, USA	17149-1-AP	1:500
CRLS1	Rabbit	Proteintech Group, USA	51055-1-AP	1:300
TAFFAZIN	Mouse	Santa Cruz Biotechnology, USA	SC-365810	1:500
GAPDH	Rabbit	Cell Signaling Technology, USA	5174S	1:8000
anti-Mouse IgG	Goat	Jackson ImmunoResearch	115-035-003	1:4000
anti-Rabbit IgG	Goat	Jackson ImmunoResearch	111-035-003	1:4000

Antibody	Source	Vendor	Cat. No	dilution
ABCA1	Rabbit	Cell signaling Technology, USA	96292S	1:1000
HMGCR	Rabbit	Thermo Fisher Scientific, USA	PA5-37367	1:1000
ABCG1	Rabbit	Proteintech Group, USA	13578-1-AP	1:1000
ABCG5	Rabbit	Proteintech Group, USA	27722-1-AP	1:800
LDLR	Rabbit	Proteintech Group, USA	10785-1-AP	1:2000
Phospho-HMGCR (Ser872)	Rabbit	Thermo Fisher Scientific, USA	BS-4063R	1:1000
SOD2	Rabbit	Cell signaling Technology, USA	13141S	1:1000
Catalase	Rabbit	Cell signaling Technology, USA	14097S	1:1000
AMPKα	Rabbit	Cell signaling Technology, USA	5832S	1:1000
Phospho-AMPKα (Thr172)	Rabbit	Cell signaling Technology, USA	2535S	1:1000
NFE2L2	Rabbit	Proteintech Group, USA	80593-1-RR	WB 1:1000<br>IF 1:500
Donkey anti-Rabbit IgG Antibody, Alexa Fluor® 594	Donkey	Thermo Fisher Scientific, USA	R37119	1:2000
