# Supplementary material for: PGC-1α repression dysregulates lipid metabolism and induces lipid droplet accumulation in the retinal pigment epithelium
Source: Cell Death Dis. 2024 Jun 1;15(6):385. doi: 10.1038/s41419-024-06762-y (PMC11144268; doi:10.1038/s41419-024-06762-y)
Supplement: Supplementary file 2 — Uncropped Western Blots [file 41419_2024_6762_MOESM2_ESM.pptx]

## Slide 1
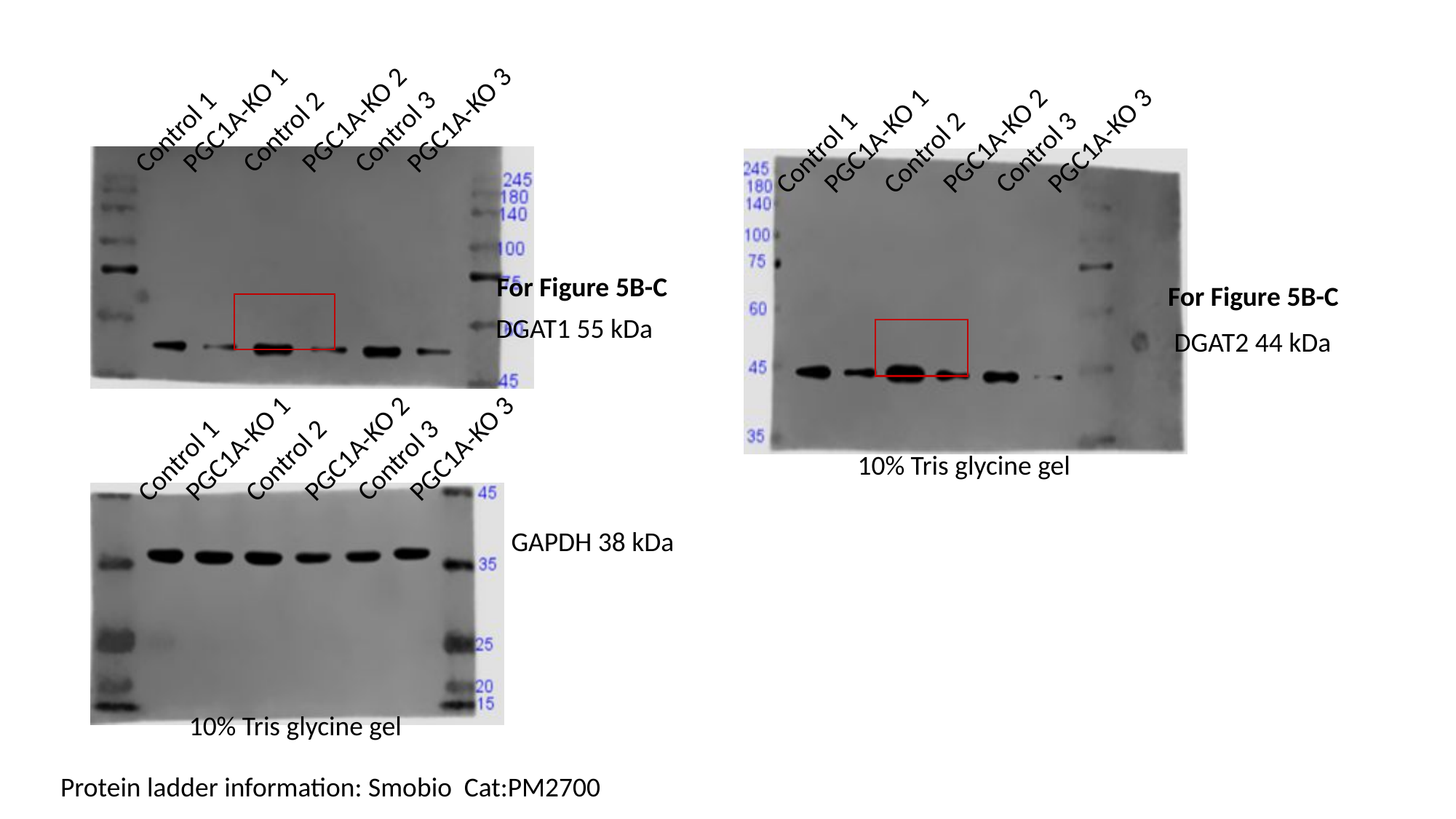

PGC1A-KO 1
PGC1A-KO 2
PGC1A-KO 3
Control 3
Control 1
Control 2
PGC1A-KO 1
PGC1A-KO 2
PGC1A-KO 3
Control 3
Control 1
Control 2
For Figure 5B-C
For Figure 5B-C
 DGAT1 55 kDa
 DGAT2 44 kDa
PGC1A-KO 1
PGC1A-KO 2
PGC1A-KO 3
Control 3
Control 1
Control 2
10% Tris glycine gel
 GAPDH 38 kDa
10% Tris glycine gel
Protein ladder information: Smobio Cat:PM2700

## Slide 2
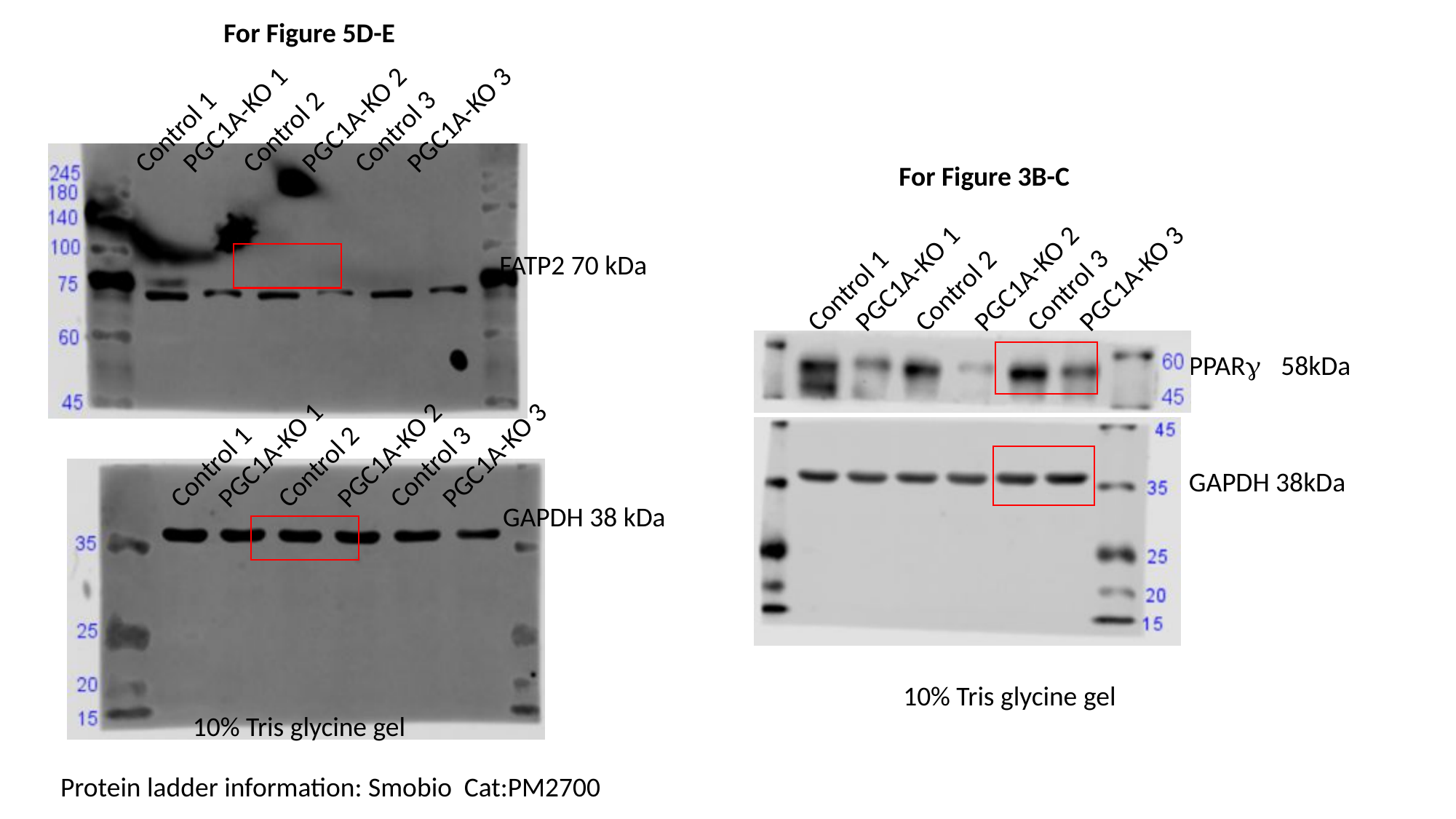

For Figure 5D-E
PGC1A-KO 1
PGC1A-KO 2
PGC1A-KO 3
Control 3
Control 1
Control 2
For Figure 3B-C
 FATP2 70 kDa
PGC1A-KO 1
PGC1A-KO 2
PGC1A-KO 3
Control 3
Control 1
Control 2
PPARg 58kDa
PGC1A-KO 1
PGC1A-KO 2
PGC1A-KO 3
Control 3
Control 1
Control 2
GAPDH 38kDa
 GAPDH 38 kDa
10% Tris glycine gel
10% Tris glycine gel
Protein ladder information: Smobio Cat:PM2700

## Slide 3
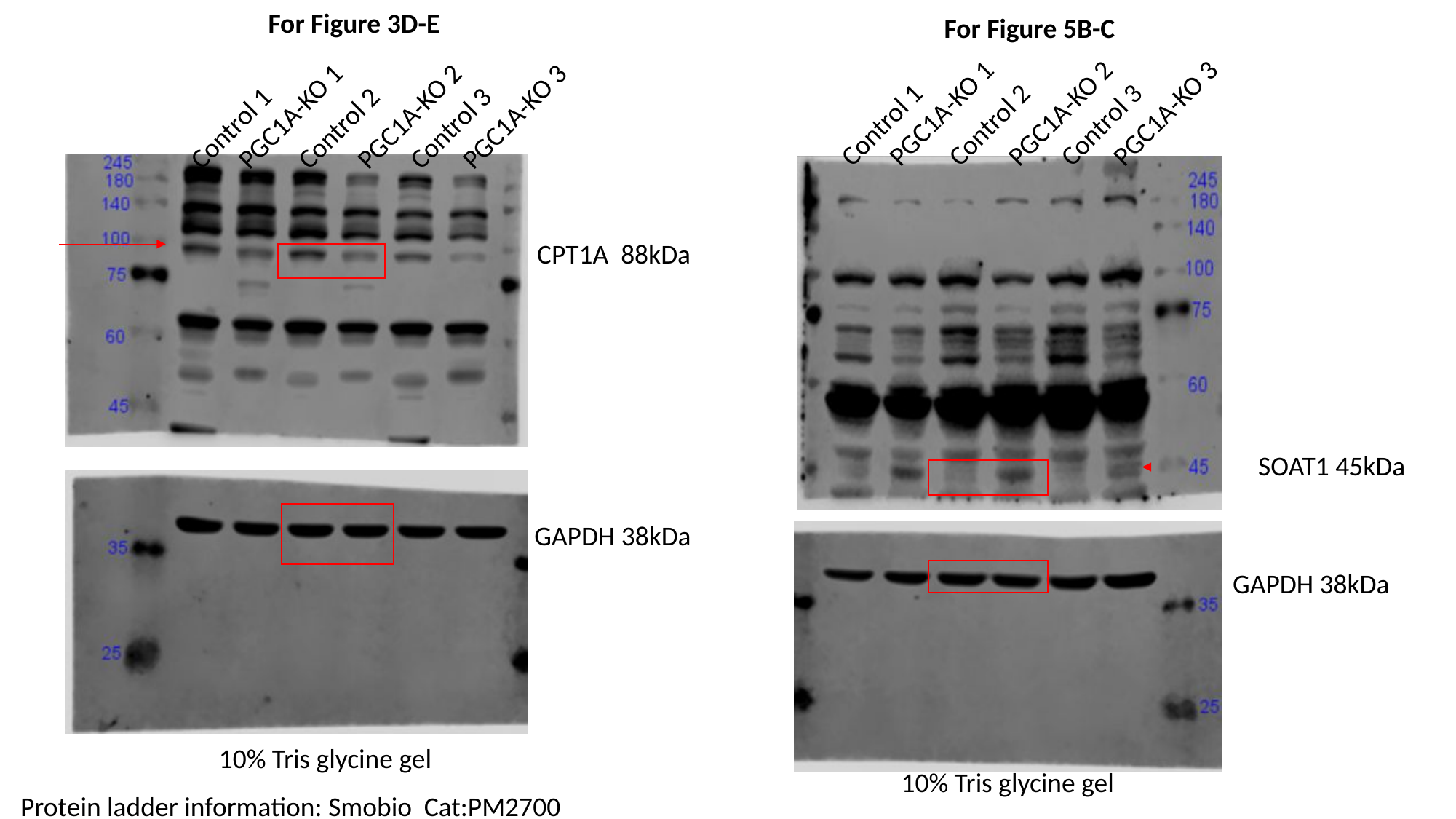

For Figure 3D-E
For Figure 5B-C
PGC1A-KO 1
PGC1A-KO 2
PGC1A-KO 3
Control 3
Control 1
Control 2
PGC1A-KO 1
PGC1A-KO 2
PGC1A-KO 3
Control 3
Control 1
Control 2
CPT1A 88kDa
SOAT1 45kDa
GAPDH 38kDa
GAPDH 38kDa
10% Tris glycine gel
10% Tris glycine gel
Protein ladder information: Smobio Cat:PM2700

## Slide 4
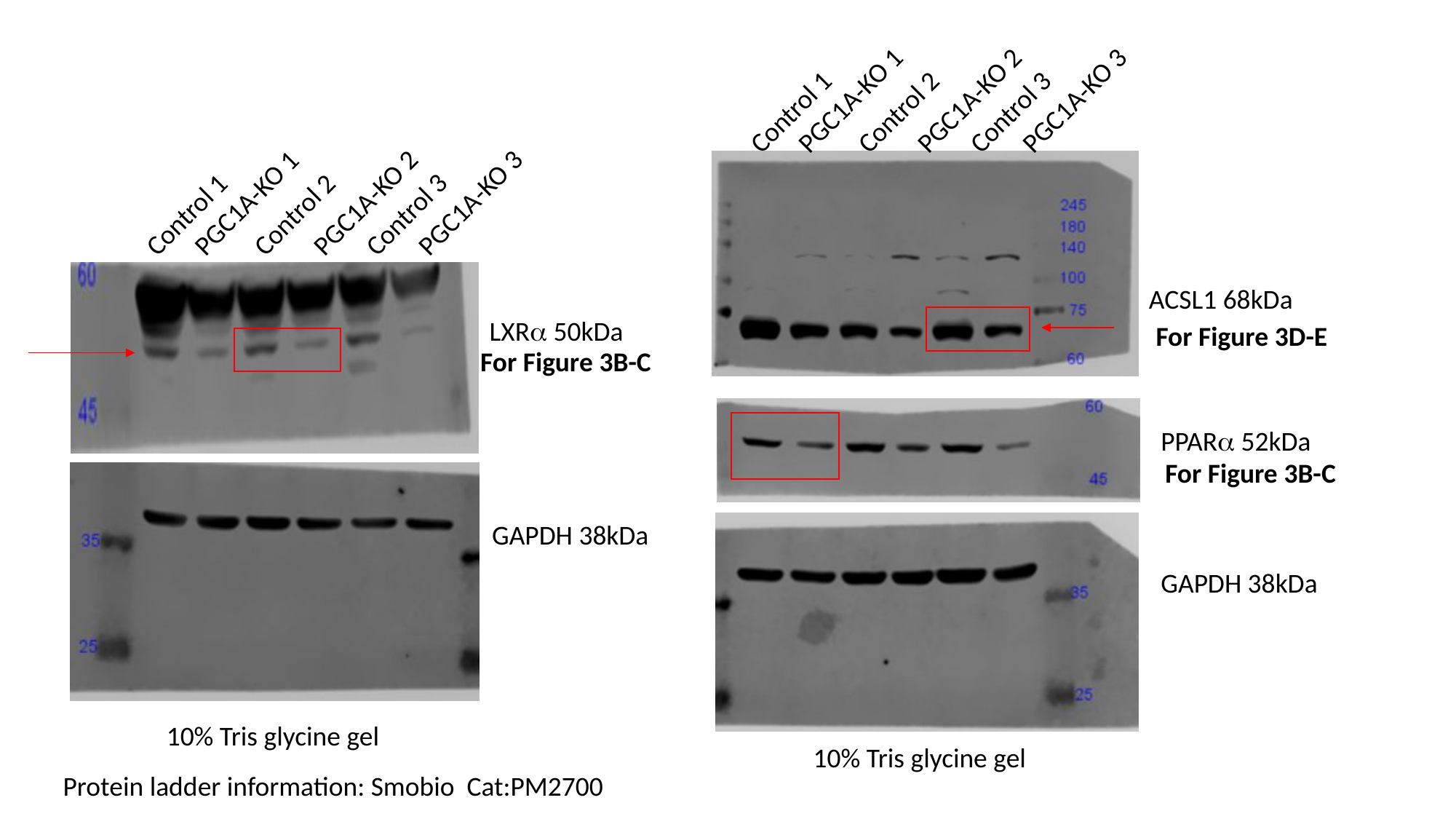

PGC1A-KO 1
PGC1A-KO 2
PGC1A-KO 3
Control 3
Control 1
Control 2
PGC1A-KO 1
PGC1A-KO 2
PGC1A-KO 3
Control 3
Control 1
Control 2
ACSL1 68kDa
LXRa 50kDa
For Figure 3D-E
For Figure 3B-C
PPARa 52kDa
For Figure 3B-C
GAPDH 38kDa
GAPDH 38kDa
10% Tris glycine gel
10% Tris glycine gel
Protein ladder information: Smobio Cat:PM2700

## Slide 5
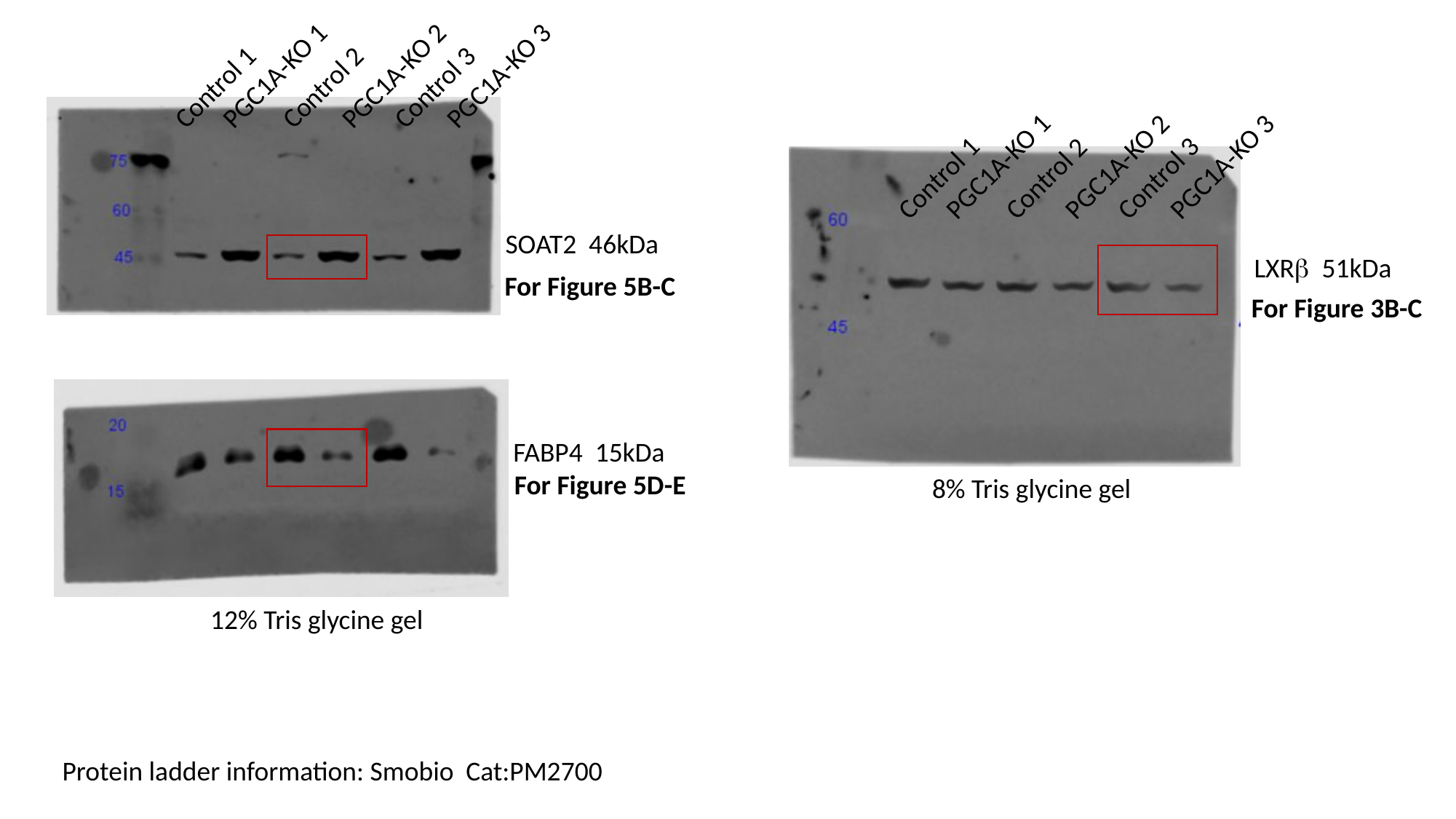

PGC1A-KO 1
PGC1A-KO 2
PGC1A-KO 3
Control 3
Control 1
Control 2
PGC1A-KO 1
PGC1A-KO 2
PGC1A-KO 3
Control 3
Control 1
Control 2
SOAT2 46kDa
LXRb 51kDa
For Figure 5B-C
For Figure 3B-C
FABP4 15kDa
For Figure 5D-E
8% Tris glycine gel
12% Tris glycine gel
Protein ladder information: Smobio Cat:PM2700

## Slide 6
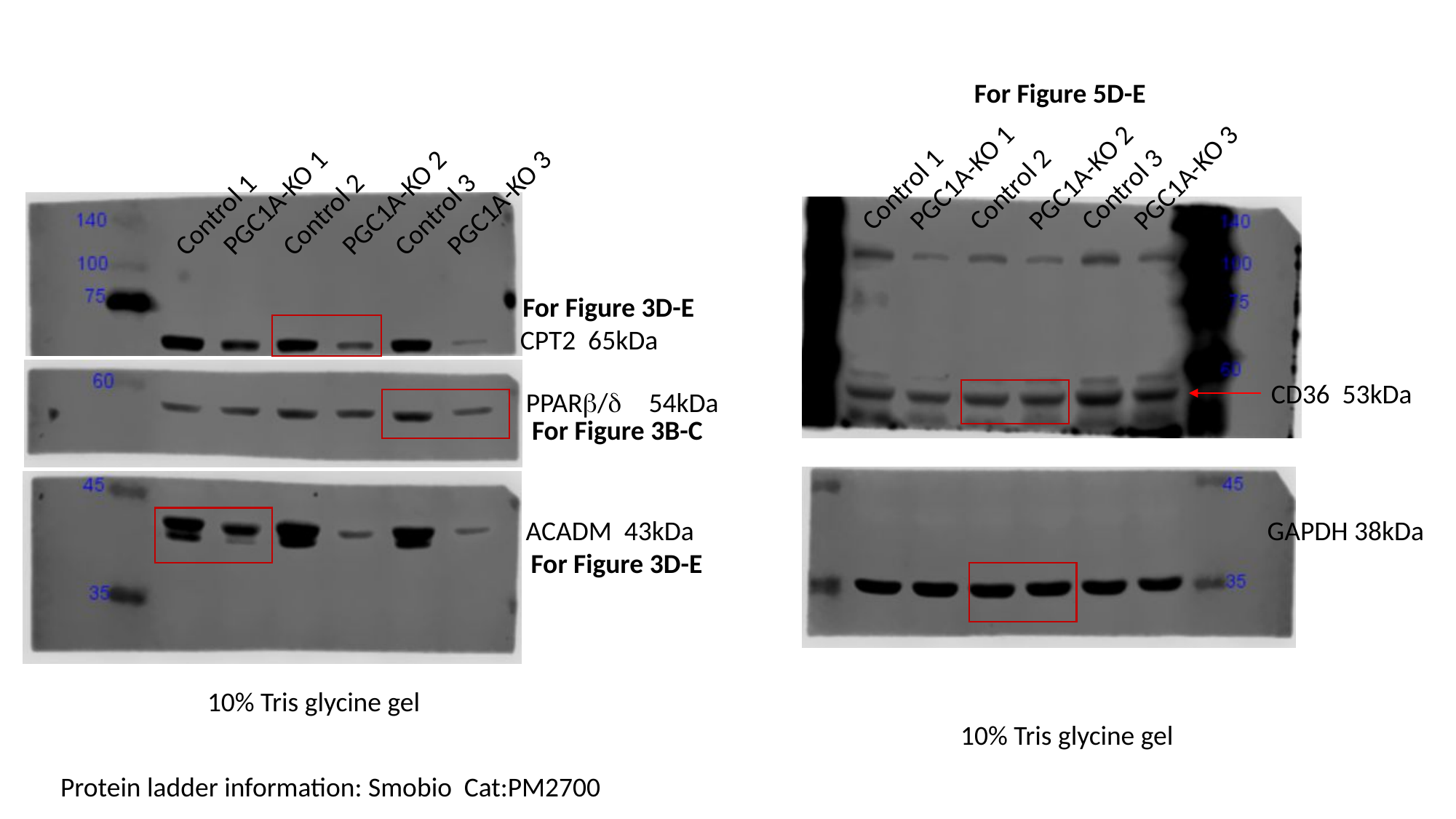

For Figure 5D-E
PGC1A-KO 1
PGC1A-KO 2
PGC1A-KO 3
Control 3
Control 1
Control 2
PGC1A-KO 1
PGC1A-KO 2
PGC1A-KO 3
Control 3
Control 1
Control 2
For Figure 3D-E
CPT2 65kDa
CD36 53kDa
PPARb/d 54kDa
For Figure 3B-C
ACADM 43kDa
GAPDH 38kDa
For Figure 3D-E
10% Tris glycine gel
10% Tris glycine gel
Protein ladder information: Smobio Cat:PM2700

## Slide 7
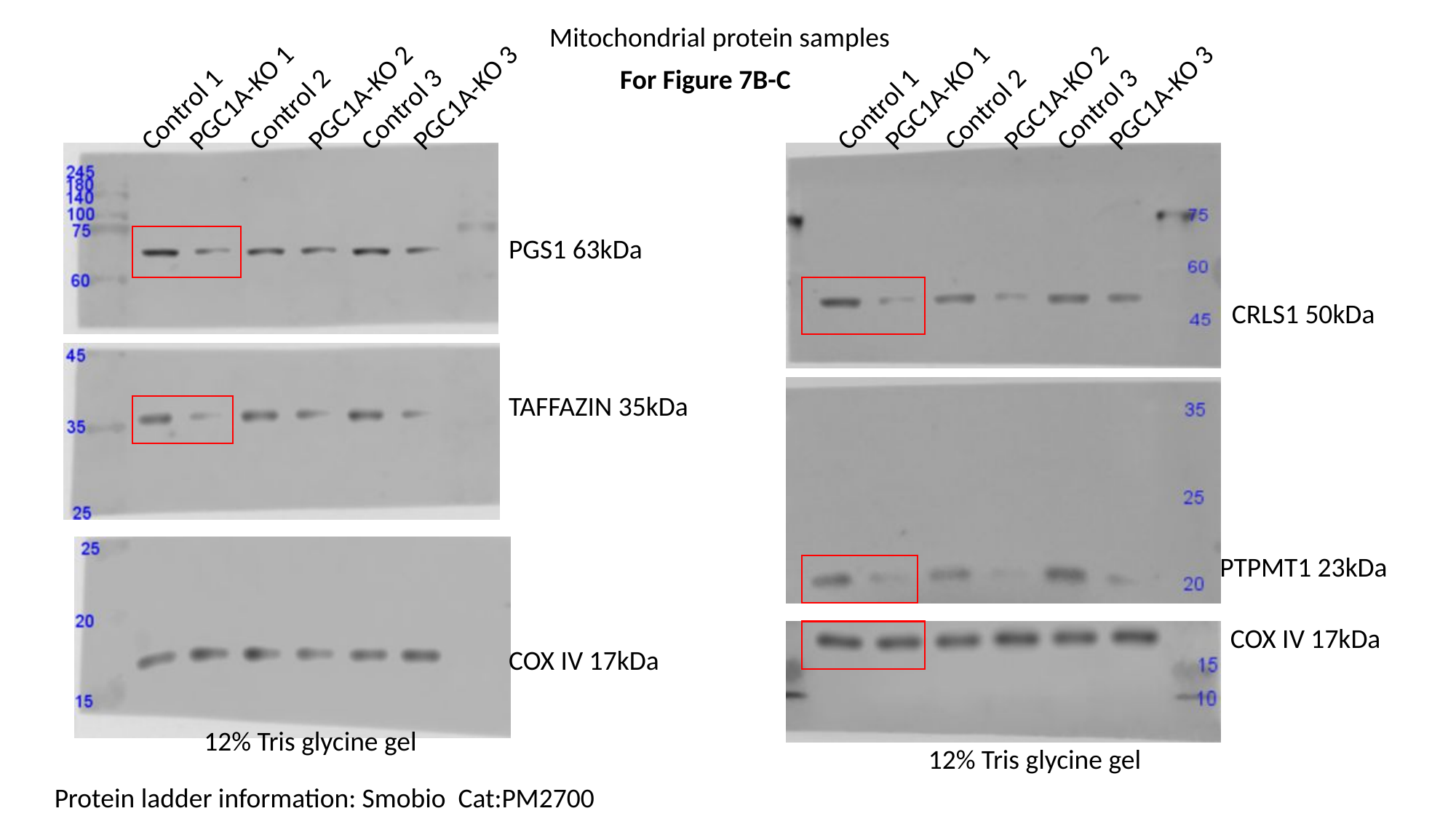

Mitochondrial protein samples
For Figure 7B-C
PGC1A-KO 1
PGC1A-KO 2
PGC1A-KO 3
Control 3
Control 1
Control 2
PGC1A-KO 1
PGC1A-KO 2
PGC1A-KO 3
Control 3
Control 1
Control 2
PGS1 63kDa
CRLS1 50kDa
TAFFAZIN 35kDa
PTPMT1 23kDa
COX IV 17kDa
COX IV 17kDa
12% Tris glycine gel
12% Tris glycine gel
Protein ladder information: Smobio Cat:PM2700

## Slide 8
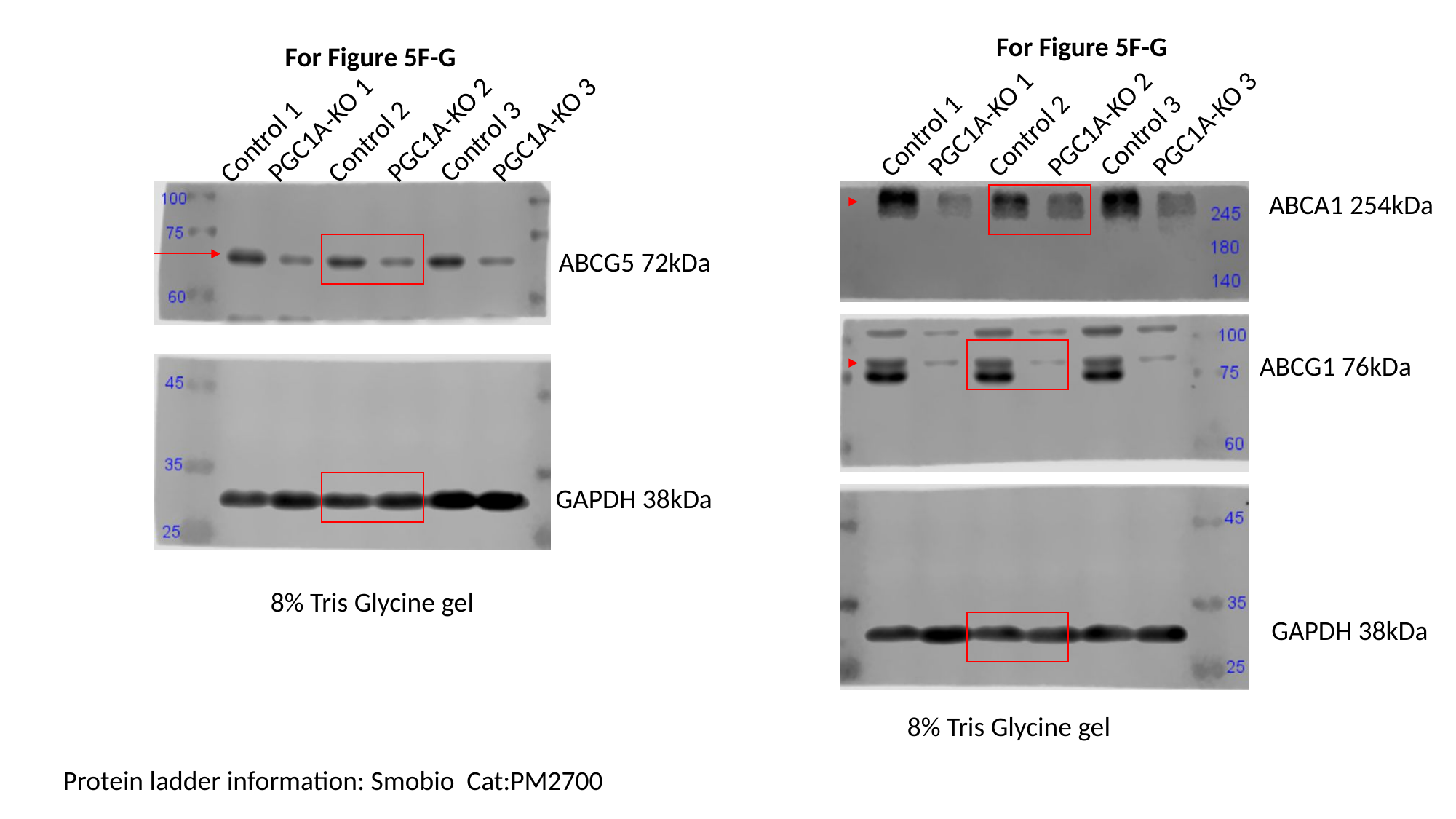

For Figure 5F-G
For Figure 5F-G
PGC1A-KO 1
PGC1A-KO 2
PGC1A-KO 3
Control 3
Control 1
Control 2
PGC1A-KO 1
PGC1A-KO 2
PGC1A-KO 3
Control 3
Control 1
Control 2
ABCA1 254kDa
ABCG5 72kDa
ABCG1 76kDa
GAPDH 38kDa
8% Tris Glycine gel
GAPDH 38kDa
8% Tris Glycine gel
Protein ladder information: Smobio Cat:PM2700

## Slide 9
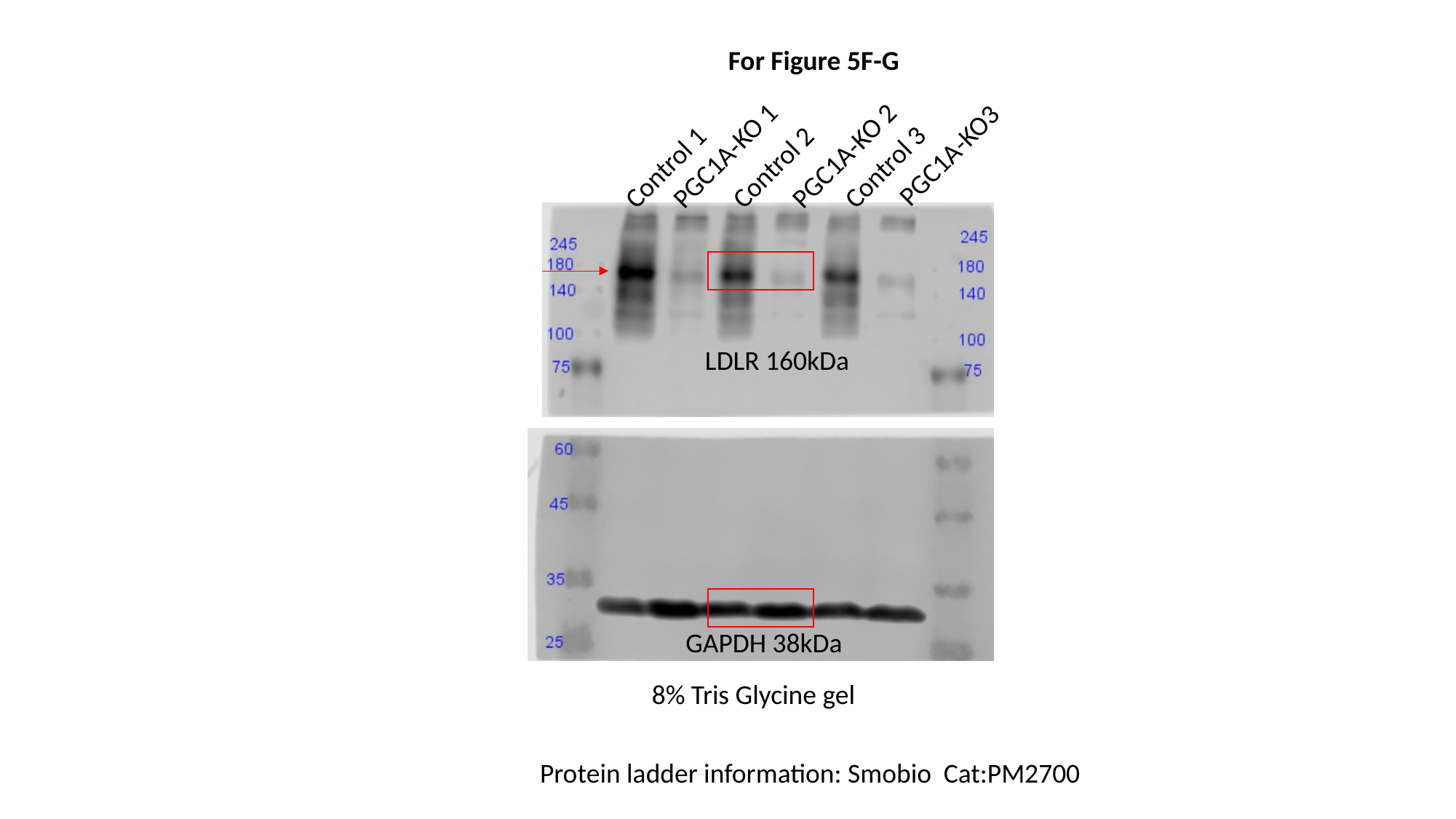

For Figure 5F-G
PGC1A-KO 1
PGC1A-KO 2
PGC1A-KO3
Control 3
Control 1
Control 2
LDLR 160kDa
GAPDH 38kDa
8% Tris Glycine gel
Protein ladder information: Smobio Cat:PM2700

## Slide 10
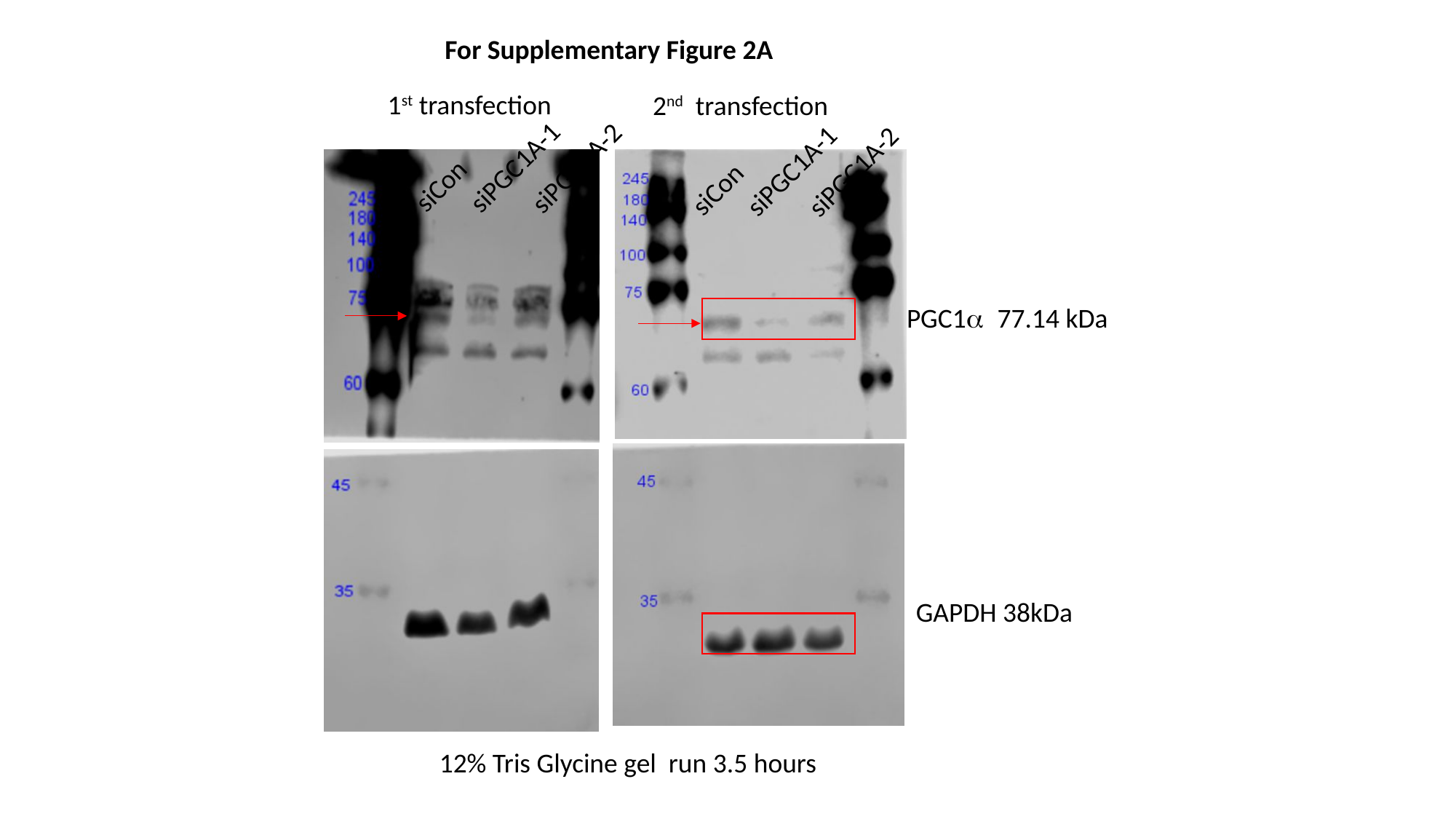

For Supplementary Figure 2A
1st transfection
2nd transfection
siPGC1A-1
siPGC1A-2
siCon
siPGC1A-1
siPGC1A-2
siCon
PGC1a 77.14 kDa
GAPDH 38kDa
12% Tris Glycine gel run 3.5 hours

## Slide 11
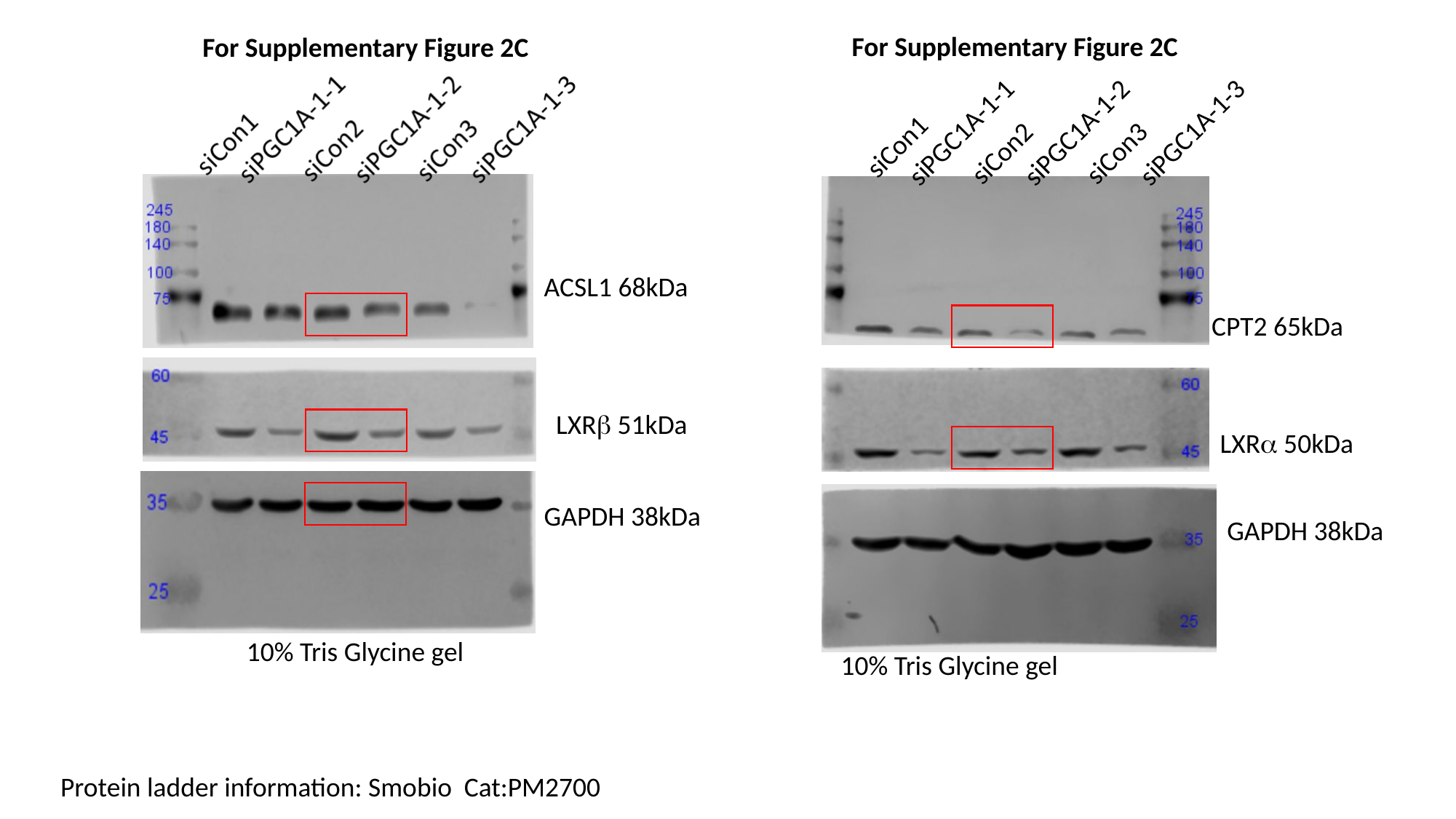

For Supplementary Figure 2C
For Supplementary Figure 2C
siPGC1A-1-1
siPGC1A-1-2
siPGC1A-1-3
siCon1
siCon2
siCon3
ACSL1 68kDa
CPT2 65kDa
LXRb 51kDa
LXRa 50kDa
GAPDH 38kDa
GAPDH 38kDa
10% Tris Glycine gel
10% Tris Glycine gel
Protein ladder information: Smobio Cat:PM2700

## Slide 12
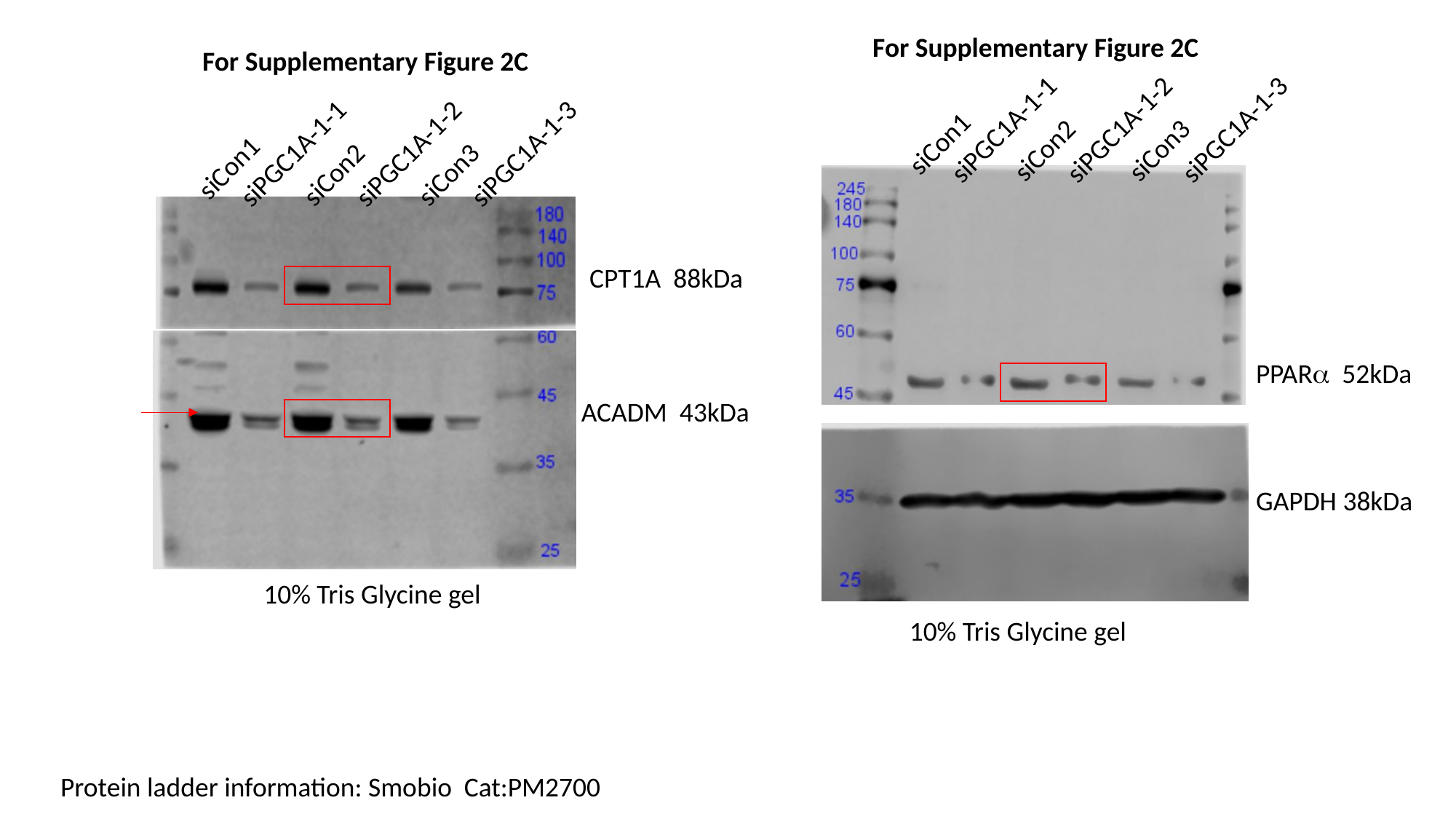

For Supplementary Figure 2C
For Supplementary Figure 2C
siPGC1A-1-1
siPGC1A-1-2
siPGC1A-1-3
siCon1
siCon2
siCon3
siPGC1A-1-1
siPGC1A-1-2
siPGC1A-1-3
siCon1
siCon2
siCon3
CPT1A 88kDa
PPARa 52kDa
ACADM 43kDa
GAPDH 38kDa
10% Tris Glycine gel
10% Tris Glycine gel
Protein ladder information: Smobio Cat:PM2700

## Slide 13
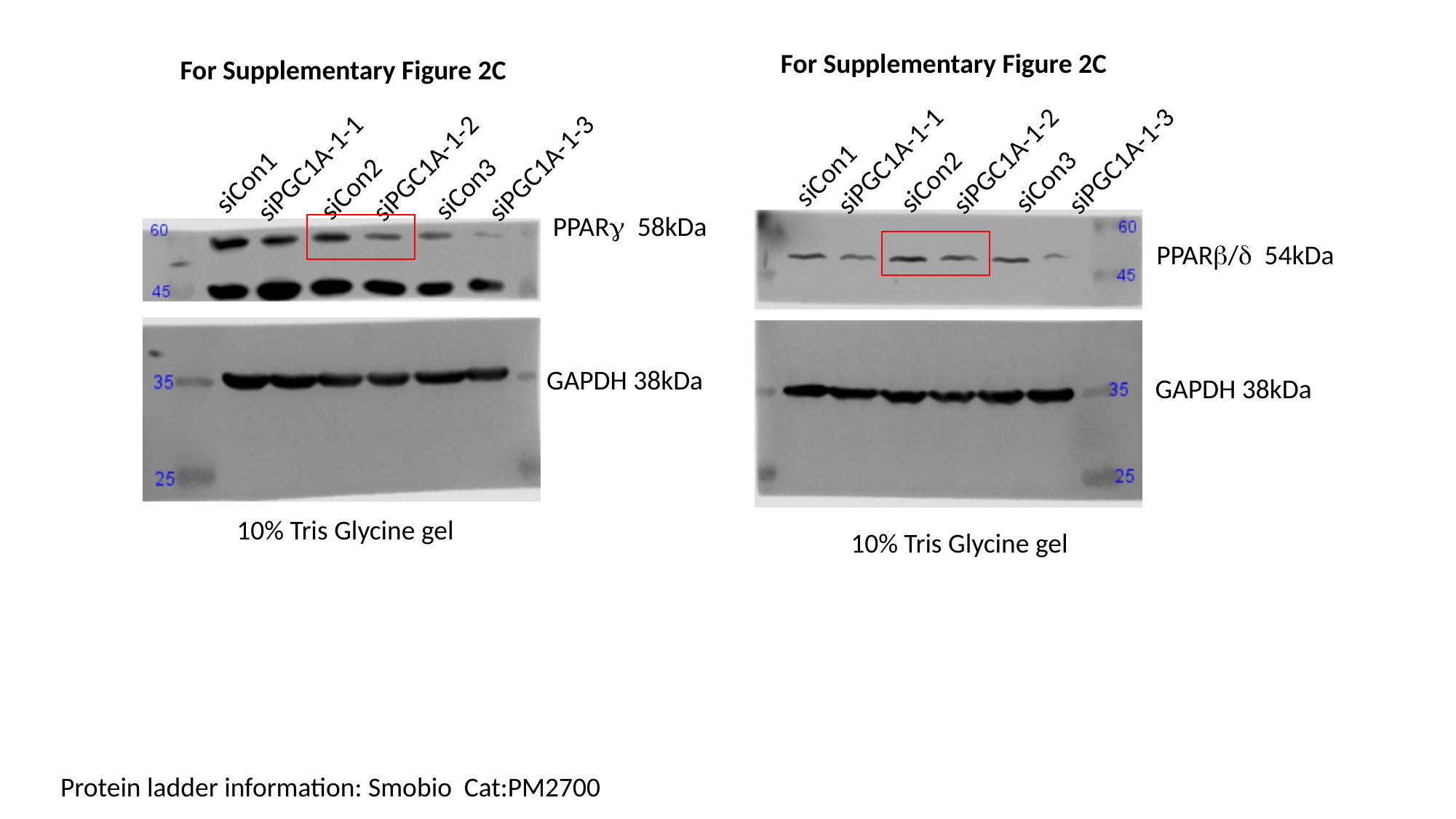

For Supplementary Figure 2C
For Supplementary Figure 2C
siPGC1A-1-1
siPGC1A-1-2
siPGC1A-1-3
siCon1
siCon2
siCon3
siPGC1A-1-1
siPGC1A-1-2
siPGC1A-1-3
siCon1
siCon2
siCon3
PPARg 58kDa
PPARb/d 54kDa
GAPDH 38kDa
GAPDH 38kDa
10% Tris Glycine gel
10% Tris Glycine gel
Protein ladder information: Smobio Cat:PM2700

## Slide 14
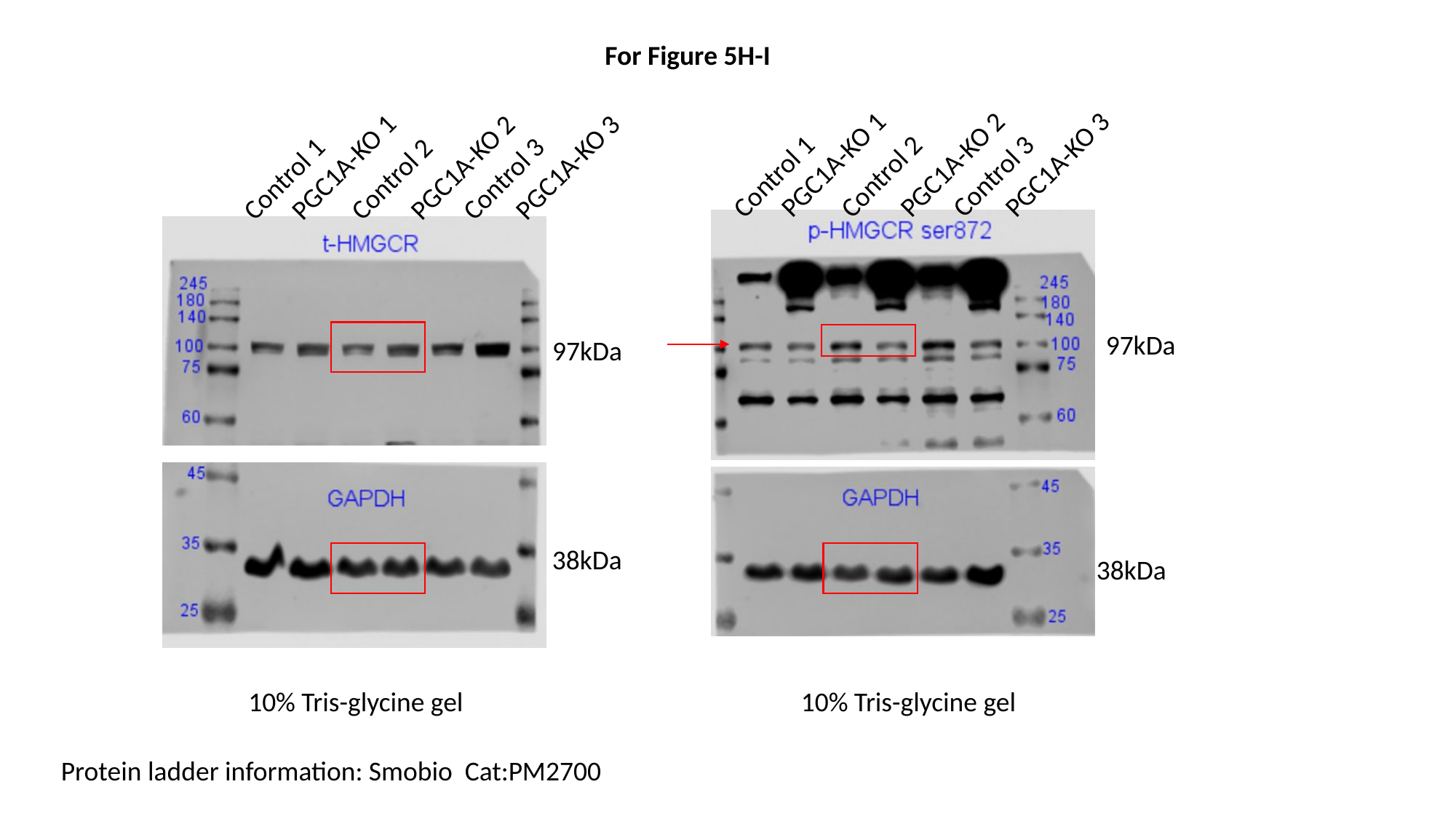

For Figure 5H-I
PGC1A-KO 1
PGC1A-KO 2
PGC1A-KO 3
Control 3
Control 1
Control 2
PGC1A-KO 1
PGC1A-KO 2
PGC1A-KO 3
Control 3
Control 1
Control 2
97kDa
97kDa
38kDa
38kDa
10% Tris-glycine gel
10% Tris-glycine gel
Protein ladder information: Smobio Cat:PM2700

## Slide 15
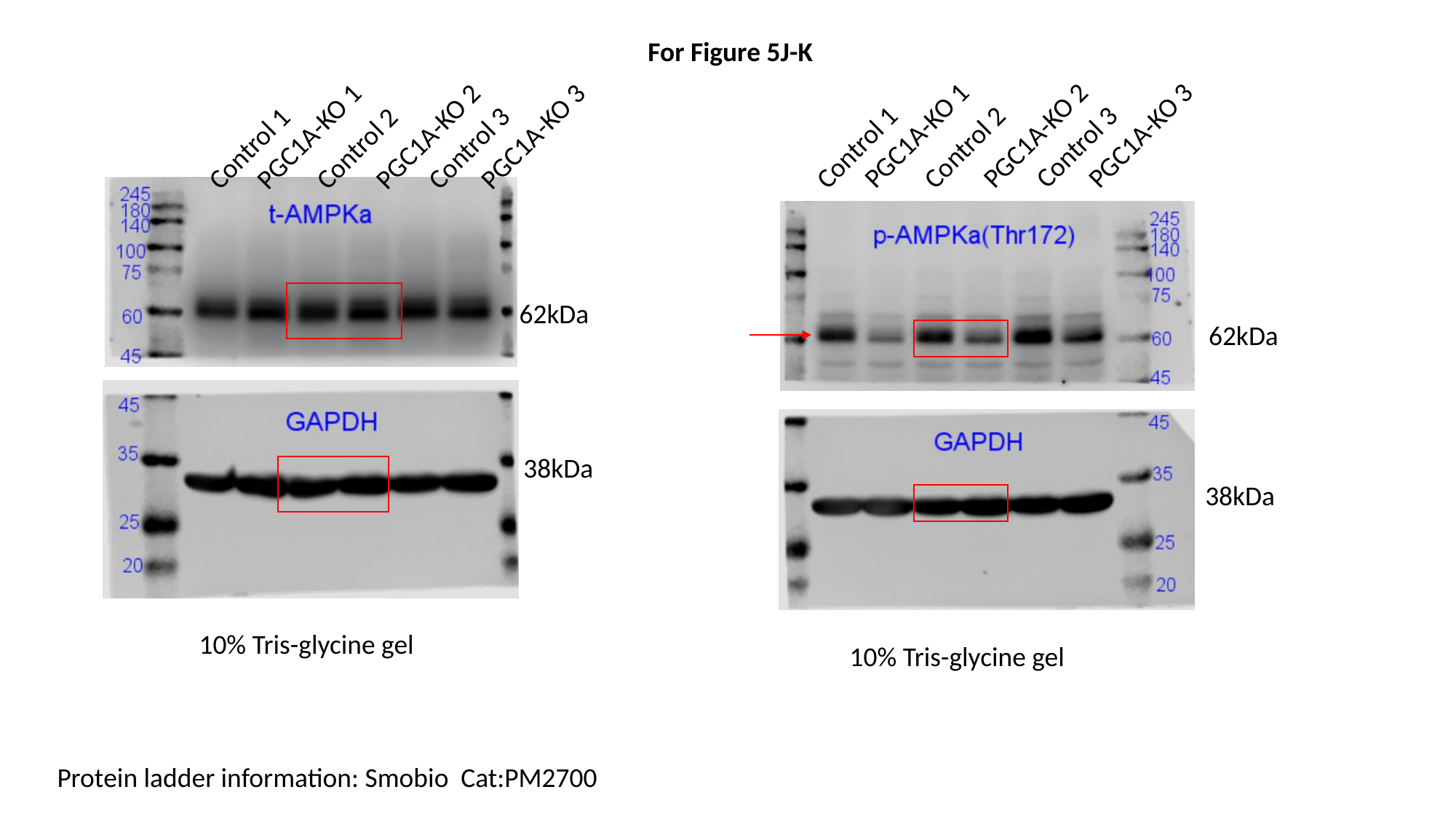

For Figure 5J-K
PGC1A-KO 1
PGC1A-KO 2
PGC1A-KO 3
Control 3
Control 1
Control 2
PGC1A-KO 1
PGC1A-KO 2
PGC1A-KO 3
Control 3
Control 1
Control 2
62kDa
62kDa
38kDa
38kDa
10% Tris-glycine gel
10% Tris-glycine gel
Protein ladder information: Smobio Cat:PM2700

## Slide 16
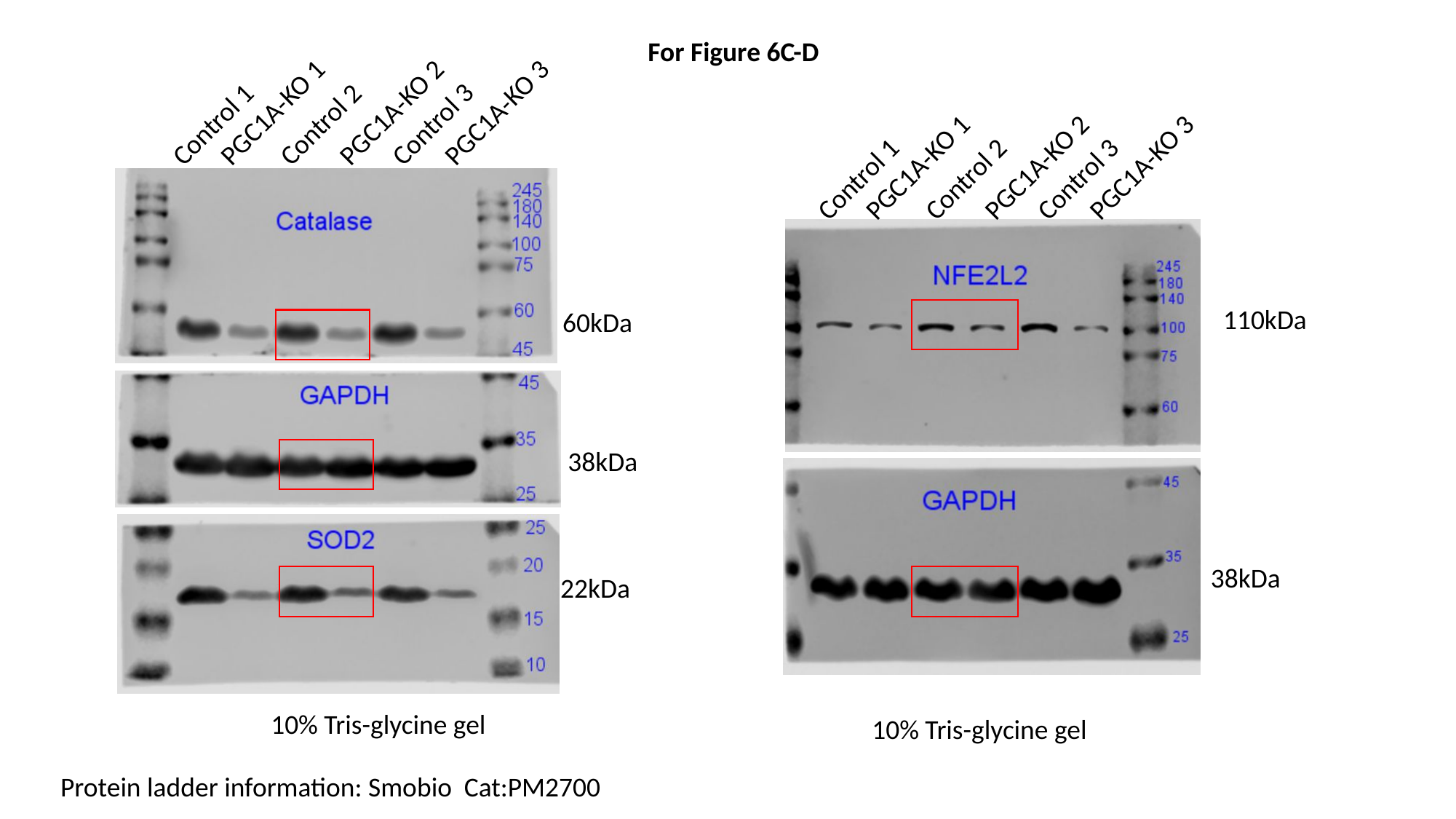

For Figure 6C-D
PGC1A-KO 1
PGC1A-KO 2
PGC1A-KO 3
Control 3
Control 1
Control 2
PGC1A-KO 1
PGC1A-KO 2
PGC1A-KO 3
Control 3
Control 1
Control 2
110kDa
60kDa
38kDa
38kDa
22kDa
10% Tris-glycine gel
10% Tris-glycine gel
Protein ladder information: Smobio Cat:PM2700

## Slide 17
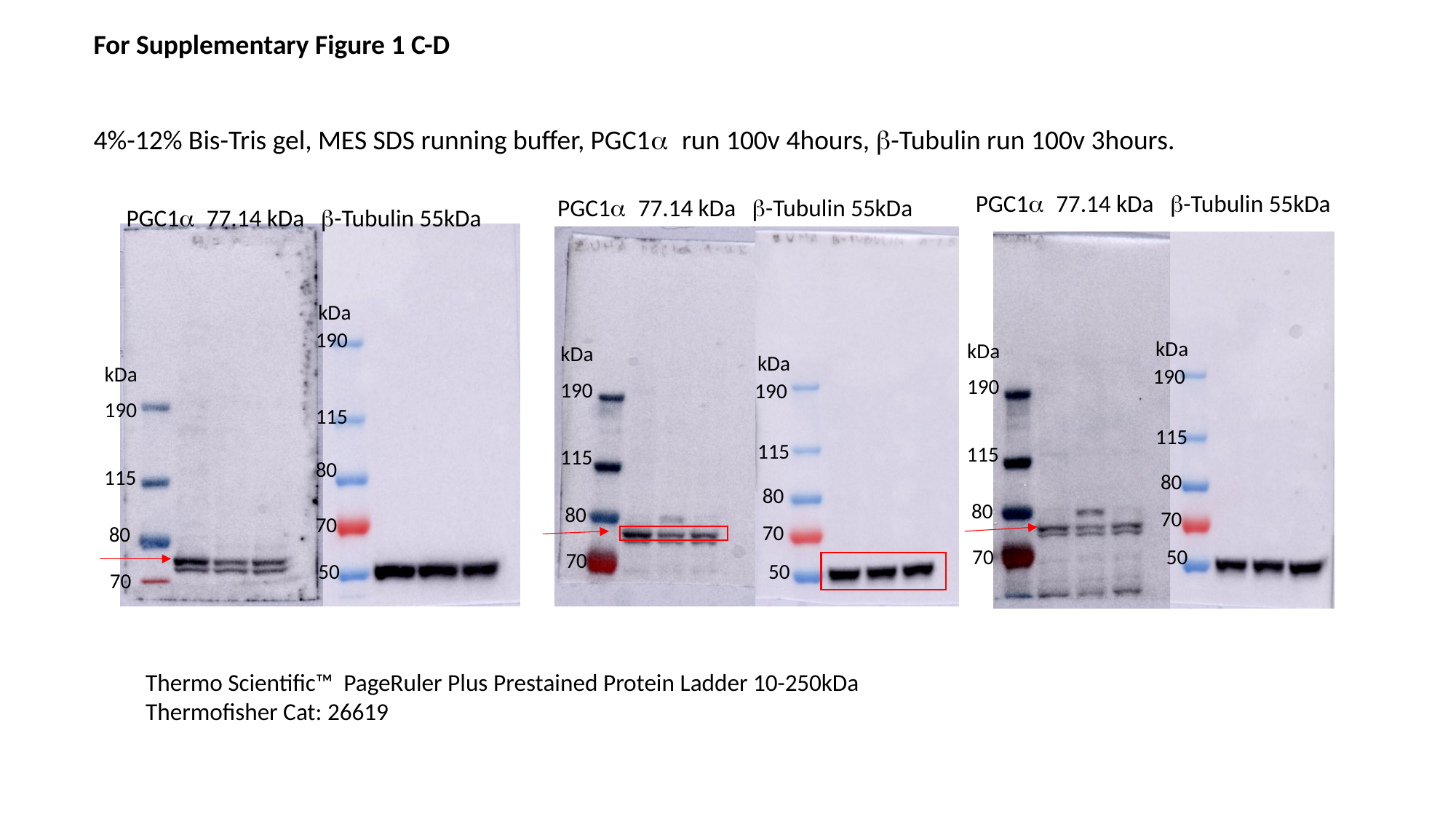

For Supplementary Figure 1 C-D
4%-12% Bis-Tris gel, MES SDS running buffer, PGC1a run 100v 4hours, b-Tubulin run 100v 3hours.
PGC1a 77.14 kDa b-Tubulin 55kDa
PGC1a 77.14 kDa b-Tubulin 55kDa
PGC1a 77.14 kDa b-Tubulin 55kDa
kDa
190
kDa
kDa
kDa
kDa
kDa
190
190
190
190
190
115
115
115
115
115
80
115
80
80
80
80
70
70
70
80
70
50
70
50
50
70
Thermo Scientific™ PageRuler Plus Prestained Protein Ladder 10-250kDa
Thermofisher Cat: 26619
